# AI‐Based D‐Amino Acid Substitution for Optimizing Antimicrobial Peptides to Treat Multidrug‐Resistant Bacterial Infection

**DOI:** 10.1002/advs.202518522

**Published:** 2026-01-14

**Authors:** Yinuo Zhao, Qingzhou Kong, Haifan Gong, Lixiang Li, Jialu Fu, Boyao Wan, Peizhu Wang, Xiaojuan Li, Yue Wang, Jinghui Zhang, Yanbo Yu, Xiaoyun Yang, Xiuli Zuo, Haina Wang, Yanqing Li

**Affiliations:** ^1^ Department of Gastroenterology Qilu Hospital of Shandong University Cheeloo College of Medicine, Shandong University Jinan China; ^2^ The Chinese University of Hong Kong Shenzhen China; ^3^ Shandong Provincial Microecological Research and Biotherapy Center Jinan China; ^4^ Shandong Provincial Clinical Research Center for Digestive Disease Jinan China; ^5^ School of Pharmaceutical Sciences Shandong University Jinan China

**Keywords:** antimicrobial peptides, antimicrobial resistance, artificial intelligence, D‐amino acids

## Abstract

D‐amino acid substitution provides an effective strategy for optimizing antimicrobial peptides (AMPs) by enhancing their stability. However, the absence of universal rules renders traditional screening methods time‐consuming and labor‐intensive, potentially leading to reduced or complete loss of activity. Here, we curated a D‐amino acid‐substituted AMP dataset from published literature and databases. We then developed ADAPT, an AI‐based tool for predicting the functional impact of D‐amino acid substitutions, and integrated it into a high‐throughput screening pipeline for AMP optimization. Of the variants obtained through this pipeline, 80% exhibited enhanced antibacterial activity. Among these, dR2‐1 showed exceptional broad‐spectrum antimicrobial activity, reduced toxicity, and substantially improved stability. Mechanistic studies confirmed a membrane‐targeting antibacterial mode of action. Furthermore, we engineered a hydrogel delivery system that effectively treated cutaneous infections in mice. Overall, our study established an AI‐based framework for D‐amino acid substitution in AMPs, enabling the efficient discovery of potent and stable candidates with enhanced clinical translation potential.

## Graphical abstract

This study constructed the first D‐amino acid antimicrobial peptide dataset and developed an AI model for efficient screening of substitution sites, with 80% of candidate peptides showing enhanced activity. The lead peptide dR2‐1 demonstrated potent antimicrobial activity in vitro and in vivo, high stability, and low toxicity. Image created in BioRender (https://BioRender.com/).

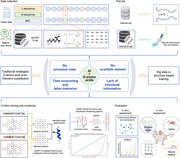

## 1 Introduction

Antimicrobial resistance has gradually increased owing to the misuse and overuse of antibiotics [1]. The rise of super bugs has led to a pressing clinical challenge with a substantial disease burden, driving an urgent need for novel antimicrobial alternatives [2, 3]. Antimicrobial peptides (AMPs) have emerged as a promising class of therapeutics against multidrug‐resistant (MDR) pathogens, offering mechanisms of action distinct from those of conventional antibiotics [4, 5]. Advances in peptide synthesis and bioengineering have also enabled the accelerated design of novel AMPs that demonstrate exceptional in vitro potency [6, 7]. However, the translation of these molecules into clinically viable drugs is often hampered by their susceptibility to proteolytic degradation [8].

Currently, strategies for improving the stability of AMPs include chemical modifications (such as the introduction of unnatural amino acids, cyclization, PEGylation, and lipidation), sequence optimization, and the conjugation with protease inhibitors [9]. Among these approaches, the incorporation of D‐amino acids into AMPs has emerged as a promising and widely used strategy to enhance proteolytic resistance [10]. Replacing enzyme‐labile L‐residues with mirror‐image D‐counterparts markedly reduces AMP susceptibility to proteolysis by impairing enzymatic recognition. However, substituting all residues with D‐amino acids as a method to completely avoid protease hydrolysis would substantially increase manufacturing costs [11]. While single or limited substitutions can improve stability under cost constraints, they may also compromise antimicrobial efficacy [12]. Moreover, because universally applicable rules for D‐amino acid substitution are lacking [13], identifying optimal positions that simultaneously maintain antimicrobial activity and confer stability remains largely empirical. This process requires iterative synthesis and microbial testing, which substantially increases both budgetary and time commitments.

To render this process more efficient and cost‐effective, we expected to employ artificial intelligence (AI) to select D‐amino acid substitution sites. However, we encountered two bottleneck issues that have similarly hindered prior efforts: (1) no publicly accessible benchmark links D‐substituted AMP sequences with corresponding antimicrobial activity assays, precluding systematic training and evaluation of computational models; and (2) the default structural oracle in the field, AlphaFold 2 [14], is trained almost exclusively on L‐amino acid proteins and consequently yields unreliable geometries for D‐chiral backbones [15]. These limitations prompted us to develop a data‐driven approach that leverages publicly available sequence‐activity data to identify suitable D‐amino acid substitutions without relying on 3D structural information.

In this study, we aimed to construct the first benchmark dataset comprising D‐substituted AMPs and develop an AI‐driven activity‐prediction framework for D‐amino acid substitutions. We aimed to systematically screen the combinatorial space of D‐amino acid substitutions across two AMP templates, indolicidin, a natural AMP derived from bovine neutrophils that is rich in tryptophan and proline and exhibits broad‐spectrum activity, and R2 [16, 17]. The top ten‐ranked D‐substituted variants were selected for synthesis and experimental validation. Wet‐lab assays revealed that 80% of these variants displayed enhanced antimicrobial activity. Among the candidates, the lead peptide dR2‐1, featuring a strategically placed single‐site D‐residue, demonstrated greater potency and stability under various conditions than the parent peptide, along with an expanded therapeutic window. Mechanistic studies confirmed its membrane‐targeting antibacterial mode of action. In a skin infection model, dR2‐1 significantly reduced bacterial burden and accelerated wound healing by applying a biocompatible hydrogel‐based delivery system. Overall, the rationale of this study was to employ an AI‐based AMP optimization framework to bridge the gap between computation‐based chemical modification and experimental realization, laying the foundation for simultaneously enhancing both antimicrobial activity and stability of AMPs to combat MDR bacterial infections.

## 2 Results

### 2.1 Overview of AI‐Based D‐Amino Acid Substitution

**FIGURE 1:**
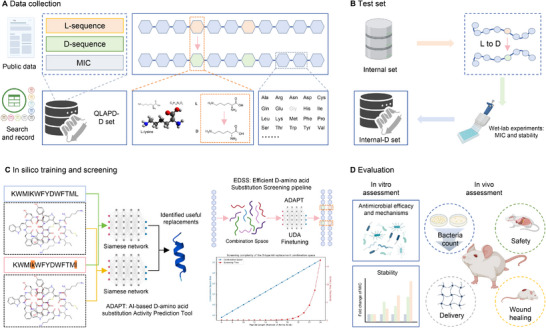
Overview of the entire research. (A) The QLAPD‐D set is derived from public literature and datasets, consisting of L‐sequences, D‐amino acid substituted sequences, and MIC values. (B) The test set is derived from previously designed L‐AMPs through D‐amino acid replacement, and includes experimentally validated MIC values, serving as an internal D‐set. (C) The in silico training and screening process utilizes a Siamese network model to identify useful sequence substitutions and optimize functionality through computational screening. (D) In vitro and in vivo experimental validation.

We assembled the first benchmark of D‐amino acid substituted AMPs from previous publications and the DBAASP database [18] (Figure 1A). A strictly held‐out wet‐lab test set produced in our laboratory was used to evaluate the models (Figure 1B). Using this dataset, we developed an AI‐based D‐amino acid substitution Activity Prediction Tool (ADAPT) and an Efficient D‐amino acid Substitution Screening (EDSS) pipeline, to identify highly effective and stable D‐amino acid substituted AMPs.

Our framework comprised three key subprocesses (Figure 1C): (1) Training the ADAPT model, a Siamese dual‐encoder module that extracts features from both sequence and SMILES representations of peptides; (2) An unsupervised domain adaptation (UDA) [19] component that fine‐tuned the model to improve generalizability across D‐modified peptides; and (3) A systematic EDSS pipeline that enumerated variants and predicted functional impact using the UDA‐fine‐tuned ADAPT model.

To rigorously evaluate the extrapolative capability of our pipeline beyond optimizing a single sequence, we applied the framework to two template AMPs, including indolicidin and R2. Ten top‐ranked D‐substituted variants for each template were synthesized for each template and validated experimentally (Figure 1D).

### 2.2 Predicting the Functional Impact of D‐Amino Acid Substitutions

To overcome the limitations of previous datasets, which primarily comprise L‐amino acid AMPs, provide limited information on D‐amino acid substitutions, and elucidate how individual D‐amino acid substitutions reshape AMP activity, we first assembled, to our knowledge, the most extensive curated collection of paired L/D AMP variants. We constructed QLAPD‐D by systematically mining the literature with two main keywords “antimicrobial peptide” and “D‐amino acid” and by screening the DBAASP database [18] for D‐AMPs and their all‐L counterparts. This dataset uniquely focused on D‐amino acid variant sequences and their activities against bacteria, providing comprehensive and relevant data to support the rational design of potent and proteolytically stable AMPs. Each pair comprised an experimentally assayed L‐form sequence and its single‐site or multiple‐site D‐amino acid counterparts. Raw activity values were normalized to minimize interlaboratory variation. Using R2 as the template, computational random substitution was performed to synthesize variants for MIC testing as the R2 test set.

To characterize the structural properties and diversity of our peptide dataset, we conducted a comprehensive statistical assessment across five dimensions. First, we quantified the similarity between the dataset templates and the antimicrobial peptide R2 as an external reference. The similarity distributions (Figure S1A) showed that the template KLWKKWKKWLK achieved the highest normalized alignment score of 0.7273 against R2, indicating potential functional proximity while preserving sequence distinctiveness. The length distributions of L‐type templates (Figure S1B) summarized the range and frequency of peptide sizes present in the dataset. The amino acid composition profiles (Figure S1C), sorted by descending frequency, highlighted the relative enrichment or scarcity of specific residues across templates. We then examined the extent of chirality modification by counting D‐type substitutions in the variants (Figure S1D). Intra‐dataset diversity was measured by the distribution of each template's maximum similarity to any other template (Figure S1E), reflecting the breadth of sequence space coverage and the level of redundancy within the collection. The distributions of the MIC ratio for the training set (2,837 sample pairs) and the R2 test set (49 sample pairs) were shown in Figure S1 F,G, respectively. In the R2 test set, the log_2_(MIC ratio) values ranged from approximately ‐2 to 6, with the majority of the data clustered between 0 and 2.

**FIGURE 2:**
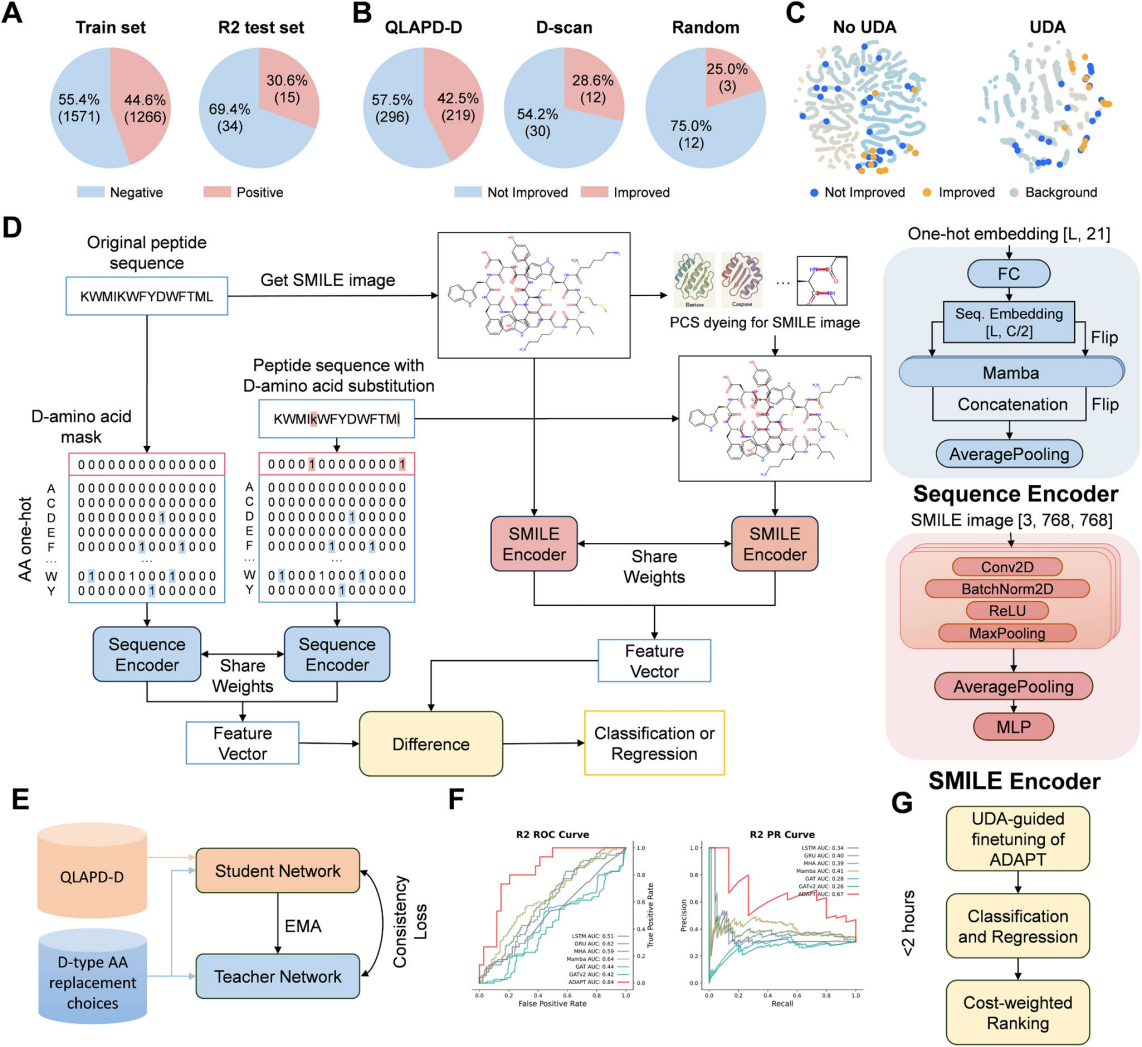
Workflow for identifying and screening D‐amino acid substitutions. (A) Distribution of the training set and the R2 test set. (B) Effects of D‐amino acid substitution on MIC. (C) UMAP projections of high–level representations for all candidate and experimentally validated R2 substitutions before and after UDA. Opaque points denote wet–lab–validated syntheses; gradient shading reflects categorical classification probability. Left, clustering corresponds to native ADAPT outputs. Right, the UDA–fine–tuned model yields clusters consistent with D–amino–acid predictions. (D) Architecture of ADAPT (AI‐based D‐amino acid substitution Activity Prediction Tool). The model comprises two Siamese branches: a Mamba branch that encodes the peptide sequence and a CNN branch that encodes the molecular image. (E) Teacher‐student training pipeline for finetuning the model. (F) Performance comparison of ADAPT with previous methods for predicting the functional impact of D‐amino acid substitutions. (G) End‐to‐end pipeline for efficiently exploring the full combinatorial space of D‐amino acid substitutions.

We visualized the distribution (Figure 2A) of the large, in silico training set and the smaller, wet‐lab‐validated test set. We also visualized the effects of D‐amino acid substitutions in AMPs based on antimicrobial activity data. In the QLAPD‐D database, 42.5% of peptides showed improved antimicrobial activity after D‐residue incorporation. We further assessed the impact of D‐amino acid substitution on antimicrobial activity using two additional strategies (Figure 2B): D‐amino acid scanning [20] and computational random substitution. Three potent AMPs previously designed [21] were selected as templates, and substitutions were performed under cost constraints by limiting the number of D‐residues to five per variant. Wet‐lab experiments revealed that only 28.6% of the variants generated by D‐amino acid scanning displayed lower MIC values. Similarly, among the variants created through random substitution, only 25% exhibited reduced MIC, confirming the time‐consuming, labor‐intensive, and inefficient nature of D‐amino acid substitution strategies.

The limitations of current 3D structure predictors when handling D‐chiral backbones [14, 15] promoted the development of our ADAPT model (Figure 2D). Rather than relying on potentially inaccurate 3D coordinates, ADAPT reformulated activity prediction as a purely sequence‐ and SMILES‐driven task. Adopting principles of Siamese representation learning in computer vision [22, 23], the network processed a native peptide and each of its D‐substituted variant in parallel, enabling direct comparison of their stereochemical perturbations. In each forward pass, the left branch was subjected to one‐hot encoding of the amino acid sequence, which a bidirectional Mamba model converted into a contextual embedding capturing long‐range epistatic effects. The right branch ingested a 2D SMILES image derived from peptide notation using RDKit. Protease‐cleavage sites (PCS) were colored based on expert knowledge [24] to highlight the feature‐level differences (Table S1). A deep convolutional neural network, ResNet‐18 [25] was used to extract topological cues, such as side‐chain packing and backbone curvature, from the SMILES image. Weight sharing across the two branches ensures that identical features are measured in both the L‐ and D‐variants, allowing emergent discrepancies to be attributed solely to chirality. A multilayer perceptron performs for the final prediction. Table S2 summarizes the results of our ablation study across sequence–only, image–only, multimodal models, and traditional machine‐learning (ML) approaches, isolating the contributions of intra–Siamese fusion and PCS dyeing to classification (AP, AUROC) and regression (PCC, KCC). Among single modalities, difference–based fusion (S–Diff: 92.82±1.13; Img–Diff: 91.32±1.56) outperformed MLP (S–MLP: 90.26±3.26; Img–MLP: 89.74±1.47) and cross–attention models (S–Att: 56.09±9.12; Img–Att: 54.58±4.09), demonstrating the advantage of simple, locality–preserving integration. Adding PCS dyeing to Img–Diff modestly increased the average score from 91.32±1.56 to 91.68±1.02, while also reducing variance. Notably, the multi‐modal weight–shared difference model (M–Diff: 93.41_±0.66_) exceeded the best image–only model by 1.73 points and the non–shared variant by 6.86 points, illustrating the value of Siamese parameter tying. Collectively, these results confirm that difference–based fusion, lightweight PCS cues, and shared multimodal representations provide consistent performance gains across both classification and regression tasks, with the multimodal approach also achieving the lowest variance among all methods.

For the Morgan fingerprint encoding scheme used in traditional ML baselines (Table S2), each molecular pair (*m*_1_, *m*_2_) used 2048‐bit Morgan fingerprints [26]. For each molecule, we computed a count fingerprint $c\left(m\right)\in R^{2048}$ and a binary fingerprint *b*(*m*) ∈ 0, 1^2048^. The pairwise feature was constructed as follows: for regression, we used the count‐difference vector Δ*c* = *c*(*m*_2_) − *c*(*m*_1_); for classification, we concatenated the count‐difference with the bitwise XOR of the binary fingerprints, yielding [Δ*c*; *b*(*m*_1_)⊕*b*(*m*_2_)].

In this study, we also proposed and experimentally evaluated an alternative approach to leverage PCS more effectively. In this method, the SMILE image was separately hinted with a D‐substitute and PCS information to generate two distinct images, which were then independently encoded using the same ResNet18 encoder. The resulting feature representations were subsequently fused through a learnable weighting parameter λ, in contrast to the original approach in which both hint types were applied to a single image and encoded in one pass. As shown in Table S3, this dual‐encoding method only achieved only performance comparable to the original ADAPT method (93.79% vs 93.95% AP, 95.07% vs 95.25% AUROC), and it exhibited substantially reduced stability, as indicated by higher standard deviations across all metrics. Additionally, this approach required twice as much VRAM and computational resources as the ADAPT baseline.

We evaluated three initialization strategies for the ResNet18 encoder weights: random initialization (no pretraining), transfer learning from the ImageMol encoder pretrained on SMILES images [27], and transfer learning from weights pretrained on the ImageNet1K natural image dataset. As shown in Table S4, all three methods demonstrated comparable performances overall, with the ImageNet1K approach showing a marginal advantage in the regression tasks (PCC: 95.53% vs 94.45% and 95.68%; KCC: 88.89% vs 87.32% and 87.48%). Notably, the ImageNet1K initialization exhibited substantially superior stability across all metrics, achieving approximately 30‐60% lower standard deviations compared with both alternative approaches. Hence, despite the domain gap between natural images and chemical structures, ImageNet pretraining provided robust low‐level visual features (e.g., edges, corners, and textures) that facilitated more stable optimization. Improved stability was particularly valuable for practical deployment, where consistent performance was critical.

In traditional peptide engineering, the primary challenge is exploring the L‐amino acid sequence space, where domain differences arise from sequence similarity variations. However, our method introduces an additional layer of complexity: beyond sequence similarity differences, the structural diversity of different peptides leads to vastly different D‐amino acid substitution patterns and their combinatorial arrangements. This creates a unique domain shift problem that goes beyond conventional sequence‐based domain differences [28, 29]. To address this issue, We cast the problem as UDA [19, 30, 31, 32] with a mean‐teacher [33] framework, which is shown in Figure 2E. We treated the labeled QLAPD‐D set as the source‐domain data and the combinatorial space T of all candidate mutants for a peptide of length *n* as the target‐domain data (i.e., $\left|T\right|=2^{n}-1$ unlabeled sequences). The mean‐teacher framework included a student network and a teacher network, both of which used the structure of the ADAPT model. The student network was trained on the source‐domain data with the supervised objectives. In contrast, the teacher network was trained on the target‐domain data via a consistency regularization, which enforced agreement between student and teacher predictions under stochastic augmentations. The teacher parameters θ_tea_ were updated by an exponential moving average (EMA) of the student parameters, stabilizing targets and propagating information from labeled to unlabeled domains. The overall loss was formulated as $L=L_{\text{sup}}+\lambda \cdot L_{\text{cons}}$, where $L_{\text{sup}}$ referred to the supervised cross‐entropy and MSE on source data, and $L_{\text{cons}}$ penalized the divergence between student and teacher outputs on target sequences. This UDA strategy leveraged the full target distribution without additional labels, thereby improving generalization across the expansive mutant space.

An ablation study of the UDA fine‐tuning strategy is shown in Table S5. To comprehensively evaluate UDA against simpler baseline approaches, we compared it with bootstrap (cross‐fold) ensemble [34, 35] and standard image augmentation [36] with ensemble methods. ADAPT with UDA fine‐tuning outperformed all alternative methods across all reported metrics on the R2 test set. Compared with the conventional cross‐fold ensemble baseline, absolute improvements were substantial, with AP increasing by 16.94 points, AUROC by 14.35 points, PCC by 25.66 points, and KCC by 24.80 points. Notably, UDA also surpassed standard image augmentation combined with an ensemble by 17.55 AP points, 18.17 AUROC points, 9.99 PCC points, and 14.99 KCC points, demonstrating that the benefits stemmed from leveraging the unlabeled target‐domain structure rather than merely increasing model diversity. Single‐modal architectures with UDA further supported this conclusion: Mamba with UDA achieved 12.60 AP point and 12.23 AUROC point improvements over the Mamba cross‐fold ensemble (63.19 vs. 50.59 AP; 83.33 vs. 71.10 AUROC), whereas ResNet18 with UDA showed 21.91 AP point and 20.87 AUROC point gains over its ensemble baseline (65.83 vs. 43.92 AP; 82.16 vs. 61.29 AUROC). These consistent improvements across different architectural backbones indicated that UDA fine‐tuning significantly improved the predictive accuracy, discrimination, and correlation with the ground truth for the ADAPT model through effective domain adaptation rather than simple ensemble or augmentation effects.

Dimensionality reduction with UMAP [37] in Figure 2C showed UMAP projections of the penultimate‐layer embeddings for the full test set (left) and the R2 case (right). In both panels, the experimentally validated syntheses were plotted as opaque points, and all other peptides were colored according to the model's predicted class probability (gradient scale). On the left, the embeddings of the native ADAPT model are separated into four well‐defined clusters that corresponded directly to their original classification labels: each cluster was dominated by peptides sharing the same predicted class, and experimentally validated examples fell squarely within their respective groupings. On the right, following UDA fine‐tuning, the embedding landscape is reorganized into two primary clusters along UMAP Dimension 1. One cluster remained enriched in native L‐amino acid peptides, whereas the other was almost exclusively composed of peptides experimentally confirmed to contain D‐amino acids. This reorganization demonstrated that UDA fine‐tuning sharpened the internal representation of the model to reflect stereochemical differences, yielding a clear separation consistent with the newly learned D‐amino acid predictions. To validate the predictive power of our computational model, we examined the correlation between predicted activity scores and experimentally determined antimicrobial efficacy. As shown in Figure S2, the predicted activity scores exhibited a strong positive correlation with MIC ratios (Pearson *r* = 0.7475, 95% CI: 0.5904–0.8500, *p* < 0.001), where MIC ratios represent the geometric mean of minimum inhibitory concentrations across all tested bacterial species. This significant correlation demonstrates that our prediction model accurately captures the structure‐activity relationship of D‐amino acid‐substituted peptides and can effectively guide the rational design of peptides with improved antimicrobial properties.

Rigorous benchmarking against the strongest published baselines (Figure 2F) showed that ADAPT outperformed all competitors on average. Specifically, the LSTM [38]. GRU [39], MHA [40], and Mamba [41] used one‐hot sequence embeddings, whereas GAT [42] and GATv2 [43] processed the molecule graph of sequence. All baselines were formulated for the siamese learning task [44]. These results underscore the value of jointly modeling the sequence context and the SMILE picture.

### 2.3 Efficient D‐Amino Acid Substitution Screening Pipeline

We implemented the end‐to‐end Efficient D‐amino acid Substitution Screening (Figure 2G, EDSS), which enumerated every mutant choice by replacing any subset of L‐residues in a peptide with their D‐enantiomers. For a peptide of length *n*, the search space comprised 2^*n*^ − 1 unique sequences (Figure S3). In this pipeline, ADAPT is a Siamese network that provides activity scoring for D‐type peptides by performing classification and regression tasks. Specifically, **ADAPT‐CLS** is a binary classifier that reports whether a given L→D mutant was predicted to surpass its all‐L parent in antimicrobial activity. The network was trained with a cross‐entropy loss on QLAPD‐D. **ADAPT‐REG** is a regression model that estimates the quantitative activity change, defined as Δlog_2_MIC = log_2_(MICmut/MICparent). The model was trained on QLAPD‐D with a mean‐squared‐error loss. We used the aforementioned UDA framework to further fine‐tune the ADAPT‐CLS and ADAPT‐REG models for downstream predictions.

The screening pipeline was made efficient by employing the following approaches: For any input peptide, the pipeline (i) cached the SMILE image embedding and sequence embedding of the unmodified template and (ii) constructed the D‐specific fragments in parallel on all CPU cores. The latter were multiplied by a synthesis‐cost coefficient λ=#L/n, prioritizing designs that retained more L‐residues. With the embeddings cached and CPU–GPU computations fully overlapping, a 14‐mer (16,384 mutants) was screened in less than 2 min on a single RTX 4090D; linear extrapolation predicted that every peptide up to 24 residues (16.7 million mutants) can be processed within 24 h, reducing the experimental burden by approximately three orders of magnitude compared with brute‐force wet‐lab mutagenesis.

### 2.4 Experimental Validation of Candidate AMPs

**FIGURE 3:**
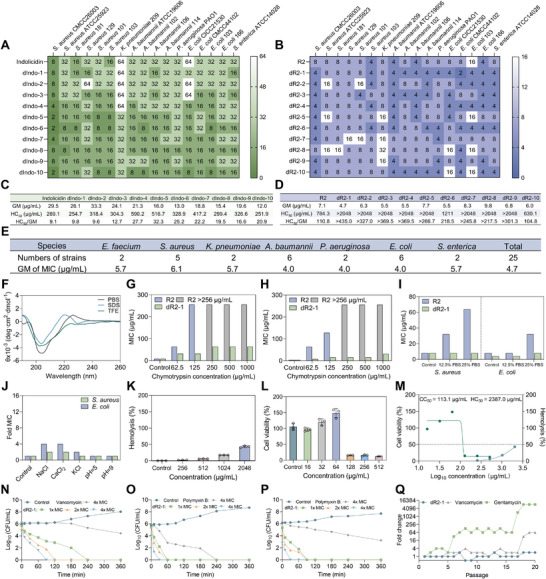
In vitro validation of candidate AMPs. (A,B) MIC of candidate AMPs. (C, D) Geometric mean (GM) of MIC, HC_50_ (50% hemolysis) and therapeutic indices (HC_50_/GM) of candidate AMPs. (E) GM of multi‐species MIC of dR2‐1. (F) Circular dichroism spectra of dR2‐1 in 10 mM PBS, 30 mM SDS, and 50% TFE environments. (G, H) Antimicrobial activity of R2 and dR2‐1 under varying concentrations of chymotrypsin against *S. aureus* (G) and *E. coli* (H). (I) Antimicrobial activity of R2 and dR2‐1 in 12.5% and 25% fetal bovine serum (FBS) environments against *S. aureus* and *E. coli*. (J) Antimicrobial activity of dR2‐1 against *S. aureus* and *E. coli* under different pH and physiological salt concentrations, pH = 5 or 9, with NaCl, CaCl_2_, and KCl concentrations of 150, 2, and 4.5 mM. (K) Hemolytic activity of dR2‐1 on red blood cells, n=3 biological replicates. (L) Cytotoxicity of dR2‐1 on 293T cells evaluated using the CCK‐8 assay, n=3 biological replicates. (M) Fitted curves for HC_50_ (50% hemolysis) and CC_50_ (50% cytotoxicity). (N) Time‐killing kinetics curves of *S. aureus* treated with different concentrations of dR2‐1 (1×, 2×, 4× MIC), using vancomycin (4× MIC) as positive control. (O, P) Time‐killing kinetics curves of *P. aeruginosa* (O) and *A. baumanii* (P) treated with different concentrations of dR2‐1 (1×, 2×, 4× MIC), using polymyxin B (4× MIC) as positive control. (Q) Resistance development assay of dR2‐1, gentamycin, and vancomycin against *S. aureus* for 20 passages. Data are presented as means ± SDs.

To validate the effectiveness of the model's prediction and selection procedures, we selected indolicidin as the initial template. We ranked all possible stereochemical variants using both ADAPT modes and combined the two lists by averaging the normalized classification probability and regressed activity score (higher values indicated superior candidates). The top ten candidates were subsequently selected for synthesis and experimental validation (Table S6). Wet‐lab results demonstrated that 90% of the peptides exhibited enhanced antimicrobial activity, and 80% showed reduced hemolytic activity. Moreover, all peptides showed improved therapeutic indices (HC_50_ / GM) (Figure 3A, C; Figure S4).

After this preliminary confirmation, we used the previously designed AMP R2 as the second template for optimization, and again selected the top ten candidates for synthesis and experimental evaluation (Table S6). The wet‐lab results showed that 70% of these peptides exhibited improved antimicrobial activity, whereas 90% peptides showed reduced hemolytic activity and improved therapeutic indices (Figure 3B,D; Figure S4). Across both template peptides, AI‐designed D‐amino acid substitutions achieved improved antimicrobial activity in 80% of the candidate variants, higher than the success rates observed in conventional strategies, including D‐amino acid scanning (28.6%) and random substitution (25%) on different template peptides. Notably, this success rate also exceeded the 42.5% baseline derived from the QLAPD‐D literature dataset, suggesting that the method may demonstrate superior and more stable performance when applied to novel peptide templates. Among these candidates, dR2‐1 displayed the greatest potency and therapeutic window and was selected for subsequent mechanistic and efficacy studies.

### 2.5 Antimicrobial and Biocompatible Properties of dR2‐1

We evaluated the antimicrobial activity of dR2‐1 against ESKAPE bacteria [45], yielding MIC values ranging from 2–8 µg mL^−1^ (Table S7). Figure 3E presents the geometric mean of the antimicrobial MIC values across the bacterial species, with dR2‐1 showing an overall geometric mean of 4.7 µg mL^−1^ (2.5 µM).

Since the secondary structure of AMPs plays a crucial role in membrane permeabilization [46], we analyzed the conformation of dR2‐1 using circular dichroism (CD) spectroscopy (Figure 3F). In phosphate‐buffered saline (PBS), 50% trifluoroethanol (TFE) and 30 mM sodium dodecyl sulfate (SDS) solutions, dR2‐1 all exhibited a predominantly β‐sheet structure.

To assess its bioactivity under physiological conditions, we examined the antimicrobial efficacy of dR2‐1 after coincubation with salt ions, serum, buffers of varying pH and enzymatic solutions. When coincubated with chymotrypsin (62.5–1,000 µg mL^−1^) for 1 h, the antimicrobial activity decreased with increasing enzyme concentration, showing a two to eightfold increase in MIC (Figure 3G,H). Notably, compared to the template peptide R2 (which completely lost activity at 250–1,000 µg mL^−1^ chymotrypsin), dR2‐1 exhibited significantly improved enzymatic stability. The MIC values fluctuated within a fourfold range in the presence of 12.5%, 25% fetal bovine serum (FBS) and different salt ions (Figure 3I,J). After incubation with solutions at pH 5 and 9 for 1 h, the MIC remained stable (Figure 3J). Together, these results demonstrated the improved physiological stability of the D‐amino acid‐modified AMP across diverse conditions relative to the parent peptide.

To elucidate the model's decision‐making process, we applied integrated‐gradient attribution [47] to both Mamba and ResNet branches (Figure S5). For the successful prediction of KWKIKWPVKWFKML with D‐Phe^10^ substitution (Figure S5A), the differential heatmap (D‐substituted minus all‐L parent) showed focused attention at the substitution site and adjacent residues, with three bonds highlighted in blue representing the chiral center (C_α_). This localized pattern indicates the model's capacity to capture stereochemistry‐dependent structural changes. Conversely, the model misclassified KKLFFKILKYL with D‐Ile^7^ substitution (Figure S5B). The differential attention map exhibited diffuse, incoherent activation patterns with conflicting signals across the backbone, suggesting failure to distinguish local chirality effects from structural noise. This may reflect confounding by long‐range conformational changes or insufficient training examples for this sequence context. This Grad‐CAM analysis represents a post‐hoc interpretation. While focused attention patterns in successful cases align with known D‐amino acid‐induced conformational changes affecting serum stability, prospective experimental validation through circular dichroism spectroscopy or NMR is required to confirm whether attention patterns truly correlate with stability‐modulating structural changes.

One major limitation for the clinical application of antimicrobial peptides is their poor biocompatibility [48]. We assessed the hemolytic toxicity and cytotoxicity of dR2‐1 and calculated the 50% hemolysis concentration (HC_50_) and 50% cytotoxicity concentration (CC_50_) to evaluate its biocompatibility. Relative to the template peptide (Figure 3A), dR2‐1 exhibited significantly reduced hemolysis (HC_50_ = 2387.0 µg mL^−1^) (Figure 3K,M). Additionally, we evaluated its cytotoxicity toward 293T cells over concentrations ranging from 16–512 µg mL^−1^, yielded a CC_50_ of 113.1 µg mL^−1^ (Figure 3L,M). These results highlighted the high biocompatibility and bacterial selectivity of dR2‐1 and demonstrated a wide therapeutic window.

Time‐killing assays were performed to further characterize the antibacterial kinetics of dR2‐1. The results showed that dR2‐1 exhibited a concentration‐dependent rapid antibacterial effect against multiple bacterial strains, with faster action compared to the antibiotic controls used (Figure 3N,O,P). Moreover, dR2‐1 demonstrated superior killing kinetics compared to R2 at all concentrations tested (Figure S6). These findings highlighted the rapid and potent antibacterial activity of dR2‐1, further supporting its potential as an effective therapeutic agent against drug‐resistant bacterial infections.

We further evaluated the potential for resistance development in *S. aureus* (Figure 3Q). Over a 20‐passage continuous‐exposure assay, no resistance to dR2‐1 emerged. In contrast, the MICs of gentamicin and vancomycin increased significantly by the 6th and 18th passages, respectively, indicating reduced bacterial propensity to develop resistance against dR2‐1.

In summary, dR2‐1 exhibited potent antimicrobial activity, enhanced physiological stability, low resistance potential, and excellent biocompatibility, rendering it a highly promising candidate for clinical development against MDR bacterial infections.

### 2.6 Antimicrobial Mechanism of dR2‐1

**FIGURE 4:**
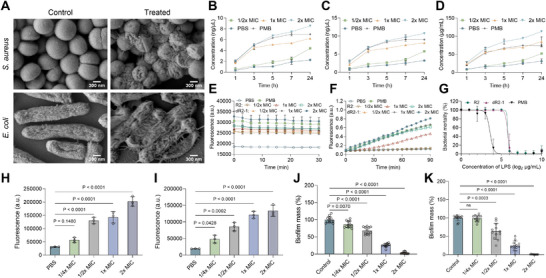
Antibacterial mechanism of dR2‐1. (A) Representative SEM images of *S. aureus* and *E. coli* treated with dR2‐1, with untreated bacteria as the control. Scale bar, 300 nm. (B, D) Detection of leaked cellular contents, including RNA (B), DNA (C), and protein (D) from *E. coli* treated with dR2‐1 (1/2×, 1×, and 2× MIC) at various time intervals. Polymyxin B (PMB) served as the positive control, n = 3. (E) Outer membrane permeability of *E. coli* treated with R2 and dR2‐1 (1/2×, 1×, and 2× MIC) determined by NPN assay. PMB served as the positive control, n = 4. (F) Cytoplasmic membrane permeability of *E. coli* treated with R2 and dR2‐1 (1/2×, 1×, and 2× MIC) determined by ONPG assay. PMB served as the positive control, n = 4. (G) Changes in the antibacterial activity of dR2‐1 against *E. coli* under different concentrations of LPS, with PMB as the positive control, n = 4. (H, I) ROS generation in *S. aureus* (H) and *E. coli* (I) treated with dR2‐1 (1/2×, 1×, and 2× MIC), with PBS treated bacteria as the control, n = 3. *P* values were determined using one‐way ANOVA with Dunnett's multiple comparison test. (J, K) Effect of dR2‐1 (1/2×, 1×, and 2× MIC) on the inhibition of biofilm formation (J) and eradication of performed biofilm (K) of *S. aureus*, n = 12. *P* values were determined using one‐way ANOVA with Dunnett's multiple comparison test. Data are presented as means ± SDs.

Previous studies have demonstrated that AMPs exert their antibacterial effects by disrupting bacterial cell membranes through cationic and hydrophobic properties [49]. To investigate the potential antibacterial mechanism of dR2‐1, we performed a series of membrane permeability‐related experiments. Scanning electron microscopy (SEM) provided direct visual evidence of the impact of dR2‐1 on bacteria (Figure 4A). Untreated *S. aureus* and *E. coli* exhibited smooth, intact surfaces, whereas dR2‐1‐treated bacteria showed severe membrane wrinkling, rupture, and cytoplasmic leakage. Membrane damage caused by AMPs or antibiotics leads to the leakage of intracellular contents (DNA, RNA, and proteins), which can be measured to assess the extent of membrane disruption [50]. Our results showed that dR2‐1 induced concentration‐dependent leakage of bacterial contents (Figure 4B,C,D).

The membrane‐disrupting activities of R2 and dR2‐1, and their LPS‐binding capabilities were further assessed using a series of assays. The NPN assay revealed a concentration‐dependent increase in fluorescence intensity following peptide treatment, indicating outer membrane penetration [50]. Notably, dR2‐1 elicited a stronger fluorescent signal than R2 at both 1× and 2× MIC, demonstrating its superior outer membrane‐disrupting capacity (Figure 4E). Consistent with these findings, the ONPG assay, which monitors cytoplasmic membrane permeability [51], showed that both peptides induced a rapid increase in inner membrane permeability at 1× and 2× MIC, reflected by a sharp increase in absorbance (Figure 4F). This enhanced potency of dR2‐1 was consistently observed in the disruption of the inner membrane as well. Lipopolysaccharide (LPS), a critical component of Gram‐negative bacterial outer membranes, acts as a key mediator in AMP‐induced membrane disruption [52]. We evaluated the LPS‐binding capacity using a competitive inhibition assay (Figure 4G). At an LPS concentration of 64 µg mL^−1^, the antibacterial activity of R2 and dR2‐1 decreased due to LPS binding, indicating the LPS affinity relevant to Gram‐negative bacteria targeting.

We further examined reactive oxygen species (ROS) levels in dR2‐1‐treated *S. aureus* and *E. coli* cells (Figure 4H,I). Dose‐dependent ROS accumulation suggested that dR2‐1 may exert antibacterial effects by entering bacterial cells and inducing oxidative stress. Biofilm formation is an important contributor to bacterial resistance [53], and AMPs have the advantage of antibiofilm effects [54]. dR2‐1 effectively suppressed *S. aureus* biofilm formation at concentrations as low as 1/4× MIC, and eradicated preformed biofilm at concentrations as low as 1/2× MIC (Figure 4J,K), indicating its potential for treating chronic biofilm‐associated infections.

### 2.7 In Vivo Antibacterial Efficacy and Toxicity of dR2‐1

**FIGURE 5:**
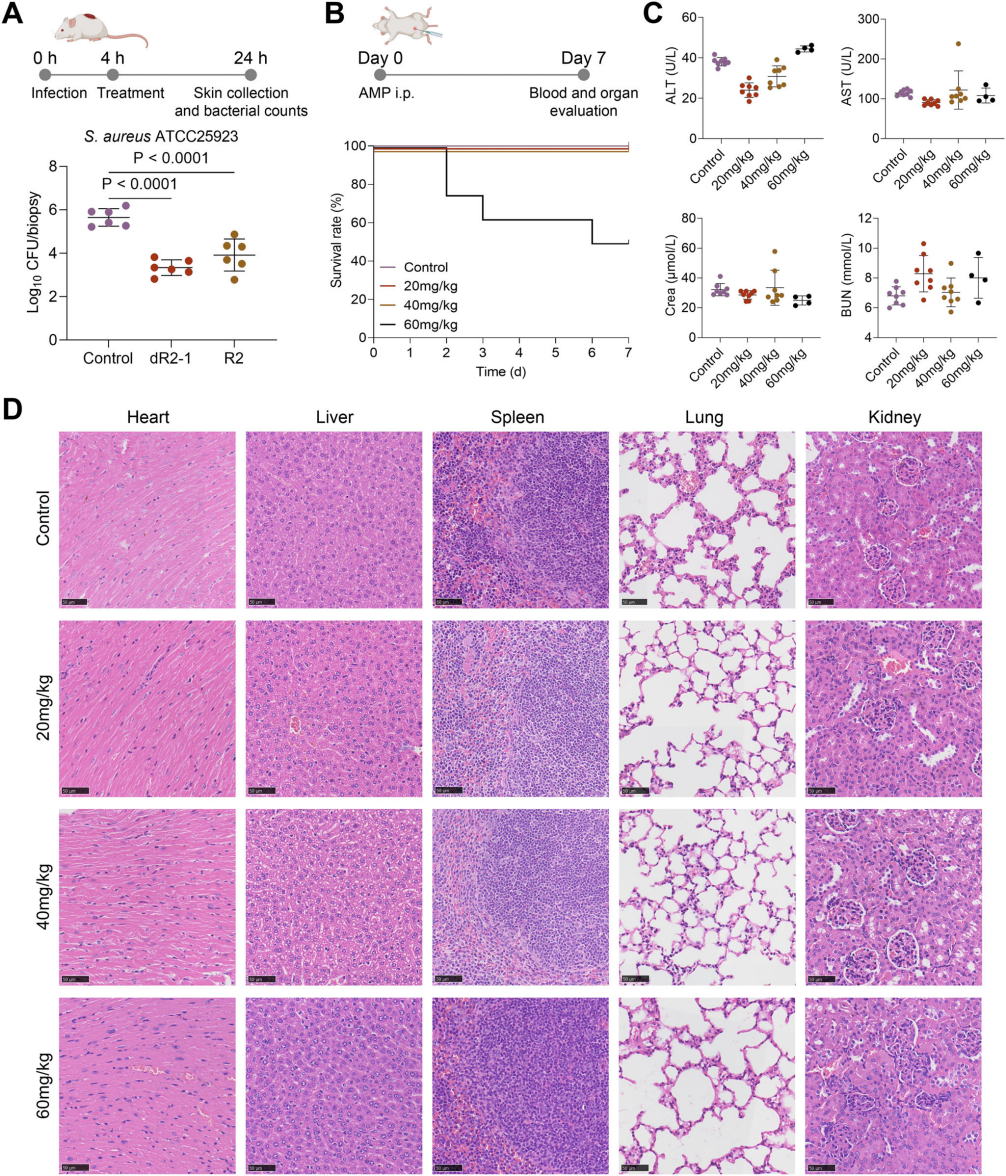
In vivo antibacterial efficacy and safety assessment of dR2‐1. (A) Schematic diagram of the in vivo antibacterial efficacy assessment model. Bacterial load of skin tissue after treatment of PBS, dR2‐1, or R2 (7.5 mg kg^−1^), n = 6. *P* values were determined using one‐way ANOVA with Dunnett's multiple comparison test. (B) Schematic diagram of the in vivo safety assessment model and survival curves of mice following a single intraperitoneal injection of PBS or dR2‐1 (20, 40, 60 mg kg^−1^), n = 8. (C) Blood biomarkers (ALT, AST, Crea, and BUN) of surviving mice at 7 days post‐injection. Data are presented as means ± SDs. (D) Representative H&E staining histopathological images of heart, liver, spleen, lung, and kidney tissues from surviving mice at 7 days post‐injection. Scale bar, 50 µm.

We used a mouse wound infection model treated with *S. aureus* to evaluate the in vivo efficacy of the AMPs (Figure 5A). A single dose of dR2‐1 or R2 (7.5 mg kg^−1^) was administered after infection. One day after infection, the control group exhibited a bacterial load of approximately 10^5^ CFUs. Both dR2‐1 and R2 significantly reduced bacterial burden. Specifically, dR2‐1 reduced the bacterial load by 2.311 (95% CI: 1.567–3.056) log units compared to the control group treated with PBS, while R2 achieved a reduction of 1.731 (95% CI: 0.9863‐2.475) log units. Further dose‐response studies of dR2‐1 showed a trend toward a progressive reduction in bacterial load with higher single doses (Figure S7A). In the multi‐dose regimen, twice‐daily administration of dR2‐1 at 10 and 20 mg kg^−1^ resulted in bacterial load reductions of 1.987 (95% CI: 1.054‐2.920) log units and 2.818 (95% CI: 1.885‐3.751) log units, respectively, compared to the control group (Figure S7B).

To evaluate in vivo toxicity, we administered intraperitoneal injections of 20, 40, and 60 mg kg^−1^ dR2‐1 to BALB/c mice. The seven‐day survival rate was 50% in the 60 mg kg^−1^ group and 100% in the 20 and 40 mg kg^−1^ groups (Figure 5B). The median lethal dose (LD_50_) was determined to be 60 mg kg^−1^. Blood biochemical analysis of surviving mice after seven days showed no significant abnormalities in alanine aminotransferase (ALT), aspartate aminotransferase (AST), creatinine (Crea) or blood urea nitrogen (BUN) levels in all dose groups (Figure 5C). The histopathological examination via hematoxylin‐eosin (H&E) staining revealed no notable abnormalities, confirming the safety of the peptide in vivo (Figure 5D). In summary, results from mouse models demonstrated that dR2‐1 possessed potent therapeutic efficacy and favorable biocompatibility in vivo.

### 2.8 Preparation of Antimicrobial Hydrogels

**FIGURE 6:**
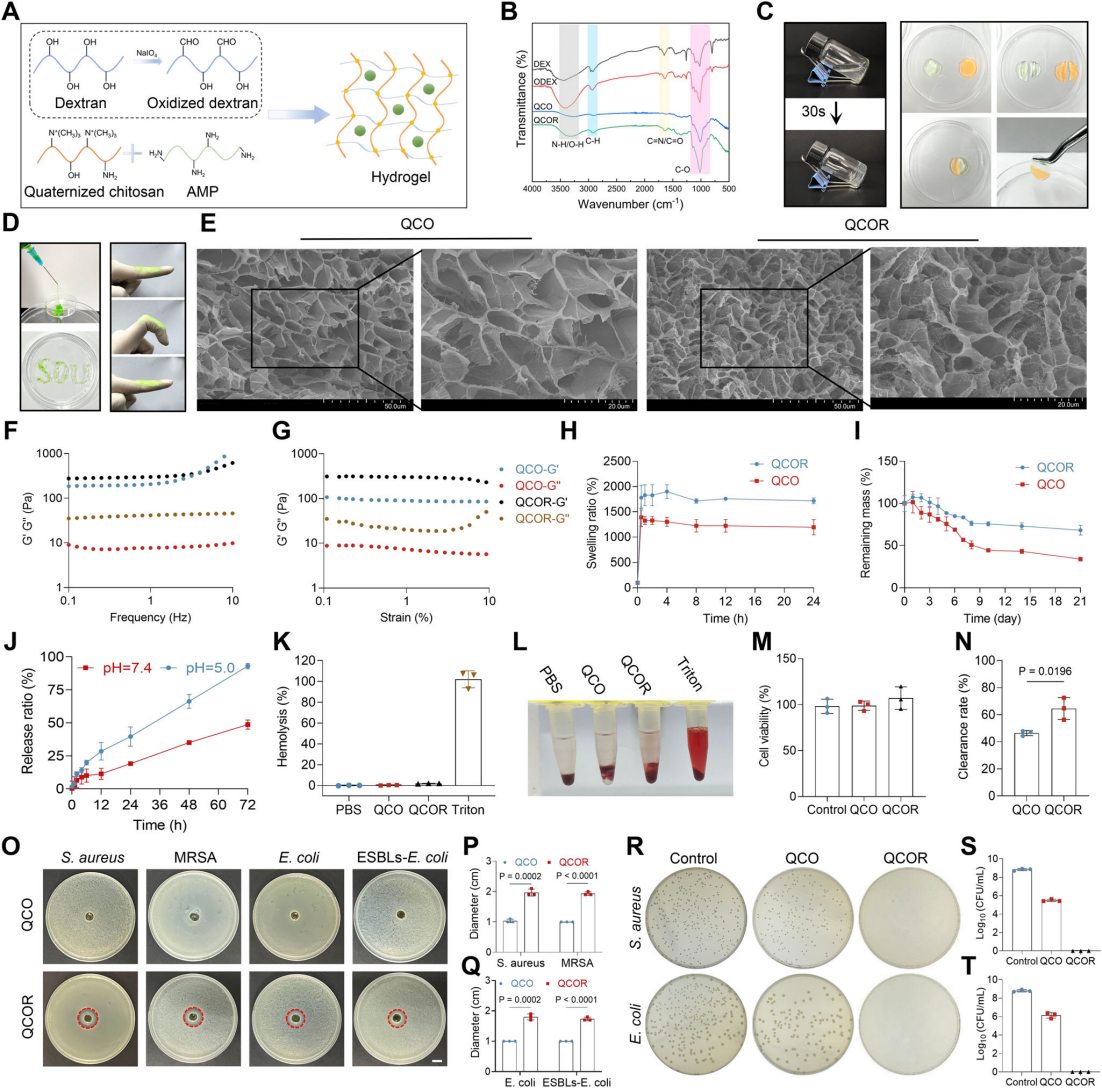
Characterization and properties of hydrogels. (A) Schematic diagram of hydrogel preparation. (B) FT‐IR spectra of hydrogels. (C) Photographic demonstration of hydrogel properties: gelation and self‐healing performance. (D) Photographic demonstration of hydrogel properties: injectable property and adhesive property. (E) Representative SEM images of QCO and QCOR hydrogels. Scale bar, 50 or 20 µm. (F) Rheological frequency sweep curves of hydrogels. (G) Rheological amplitude sweep curves of hydrogels. (H–J), Swelling (H), degradation (I), and drug release behaviors (J) of hydrogels. (K, L) Hemolytic toxicity assay of hydrogels and representative images. (M) Cytotoxicity of hydrogels on 293T cells evaluated using the CCK‐8 assay. (N) Antioxidant capacity assessment of hydrogels via ABTS radical scavenging assay, n = 3. *P* values were determined using t‐test. (O) Inhibition zones against *S. aureus*, MRSA, *E. coli* and ESBLs‐*E. coli*. The inhibition zones are shown in red dashed circles. Scale bar, 1 cm. (P, Q) The corresponding inhibition zone diameters. *P* values were determined using t‐test. (R –T) Antibacterial activity of hydrogels evaluated by colony forming unit assay and representative colony images. Data are presented as means ± SDs (n = 3 biological replicates).

Based on dR2‐1, we developed a hydrogel delivery system using quaternized chitosan (QCS) and oxidized dextran (ODEX) as the framework (Figure 6A). Fourier transform infrared spectroscopy (FT‐IR) confirmed successful ODEX synthesis and the formation of hydrogen bonds/Schiff base linkages within the hydrogel (Figure 6B). In the dextran (DEX) spectrum, characteristic peaks appeared at 3453 cm^−1^ (O‐H stretch), 2965 cm^−1^ (C‐H stretch), 1654 cm^−1^ (C = O stretch), 1442 cm^−1^ (C‐H bend), 1170 cm^−1^ (C‐O‐C stretch), 1105 cm^−1^ (C‐O stretch), 1020 cm^−1^ (C‐C stretch), and 801 cm^−1^ (C‐H bend). For ODEX, a new peak emerged at 1639 cm^−1^, corresponding to C = O stretching of the aldehyde group (‐CHO), confirming successful oxidation. The shift and intensification of the original 1654 cm^−1^ peak to 1639 cm^−1^ further verified oxidation, while peaks at 1020 and 801 cm^−1^ indicated retention of the polysaccharide backbone. In the QCO spectrum, the broad peak at 3278 cm^−1^ suggested vibrations from quaternary ammonium groups (‐N^+^(CH_3_)_3_) and hydrogen bonding, while the 1616 cm^−1^ peak (C = N) confirmed Schiff base formation between QCS and ODEX. For QCOR, the addition of dR2‐1 led to an O‐H peak at 3286 cm^−1^ (hydrogen bonding) and a C = N peak at 1636 cm^−1^ (Schiff base). Hydroxylamine hydrochloride‐methyl orange titration determined an ODEX oxidation degree of 40.5%. For the formation of hydrogel, in brief, dR2‐1 was dispersed in QCS (4% w/v) to form the QCR solution (the final concentration of dR2‐1 was 4‰w/v), then the QCR solution and the ODEX (5% w/v) solution were thoroughly mixed in equal volume to form the QCOR hydrogel. The QCOR hydrogel achieved gelated within 30 s of mixing (Figure 6C).

### 2.9 Characterization of Antimicrobial Hydrogels

Self‐healing, injectability and adhesive properties are crucial for wound applications [55]. Macroscopic self‐healing tests showed that sliced hydrogel pieces could reassemble into an intact, liftable structure, attributed to reversible Schiff base bonds (Figure 6C). The QCOR hydrogel extruded smoothly from a syringe, exhibiting excellent injectability, and adhered firmly to glove surfaces after stretching (Figure 6D). SEM revealed porous structures for both QCO and QCOR, with QCOR displaying smaller, denser pores due to dR2‐1 incorporation (Figure 6E). The solid‐like properties of hydrogels were characterized by the storage modulus (G'), while the liquid‐like properties were represented by the loss modulus (G'’). Within the frequency range of 0.1–10 Hz, both QCO and QCOR hydrogels exhibited G' >G'’, indicating viscoelastic behavior and a stable internal structure (Figure 6F). Additionally, across the strain range of 0.1%–10%, the G' and G'’ of both hydrogels remained stable. Although QCOR‐G'’ showed a slight increase at high strain levels, the overall trend demonstrated the structural stability of the hydrogels (Figure 6G). Swelling and degradation studies showed QCOR outperformed QCO, likely due to enhanced hydrogen bonding (Figure 6H,I). Both hydrogels absorbed water rapidly within 30 min, reaching equilibrium in four hours, with 24‐h swelling ratios of 11.9 (QCO) and 17.2 (QCOR).

We measured the peptide release rate in QCOR under PBS conditions with different pH values (5.0 and 7.4) (Figure 6J). The hydrogel exhibited pH‐dependent drug release kinetics, demonstrating significantly accelerated release at pH 5.0 (approximately 40% in 24 h and >90% by 72 h) compared with pH 7.4 (approximately 20% in 24 h). When applied to wounds, hydrogels come into contact with blood and tissue cells at the wound surface, necessitating hemocompatibility and cytocompatibility for practical use [56]. We evaluated the hemolytic activity of the hydrogels using a hemolysis assay (Figure 6K,L). The results demonstrated that both QCO and QCOR exhibited hemolysis rates below 5%, indicating their favorable blood compatibility. We further evaluated the cytocompatibility of the hydrogels using 293T cells. After 24 h of co‐incubation with the extraction liquids of the QCO and QCOR hydrogels, the cell viability assays indicated that both hydrogels exhibited negligible cytotoxicity toward 293T cells (Figure 6M). Additionally, we assessed the antioxidant capacity of QCO and QCOR using a 2, 2′ ‐azino‐bis (3‐ethylbenzothiazoline‐6‐sulfonic acid) (ABTS) assay kit (Figure 6N). Hence, QCOR demonstrated superior antioxidant performance compared with QCO.

S. aureus and E. coli were selected to evaluate the in vitro antibacterial properties of the hydrogels. The methods used included colony‐forming unit and inhibition‐zone assays. In the inhibition‐zone assay, QCO failed to form a noticeable inhibition zone for either the sensitive or resistant bacterial strains. However, after incorporation of dR2‐1, QCOR exhibited a clear inhibition zone (Figure 6O,P,Q). In addition, in the colony forming unit assay, after co‐incubation of the hydrogel with the bacterial suspension, QCO reduced the bacterial load compared to the control group, whereas no bacterial colonies were observed in the QCOR group, demonstrating its potent antibacterial activity (Figure 6R,S,T).

### 2.10 In Vivo Antimicrobial and Wound‐Healing Effect

**FIGURE 7:**
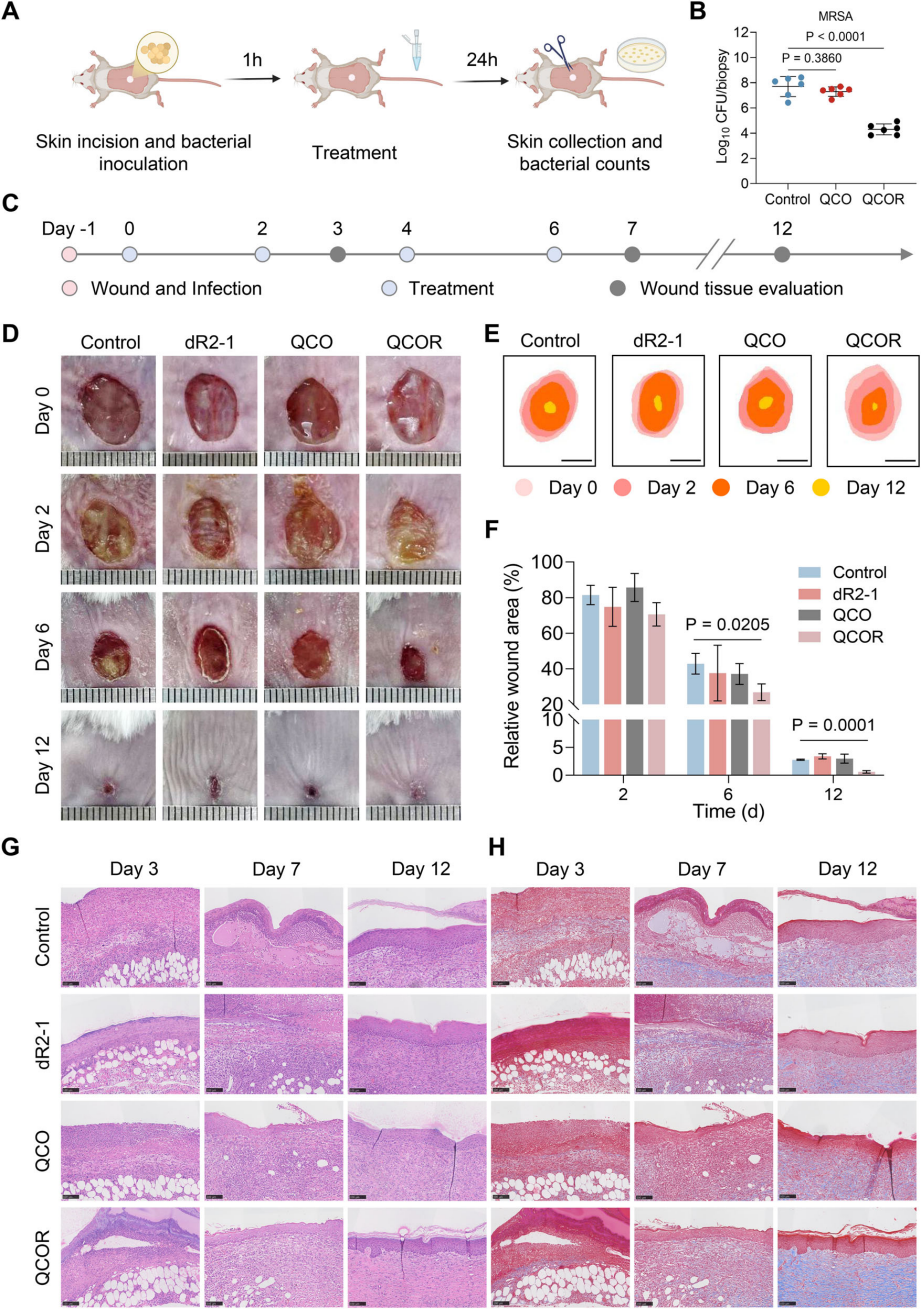
Antimicrobial hydrogel treatment promotes healing of infected chronic wounds in mice. (A) Schematic diagram of full‐thickness skin wound infection model in mice. (B) Bacterial load in infected skin tissue, n = 6. *P* values were determined using one‐way ANOVA with Dunnett's multiple comparison test. (C) Schematic illustration of hydrogel therapy for chronic wound healing. n = 6. (D) Representative wound images at days 0, 2, 6, 12. (E) Wound healing trajectory. Scale bar, 5 mm. (F) Wound healing percentage of different groups at different time points (days 2, 6, 12). *P* values were determined using t‐test. (G) Representative H&E staining images of each group at different time points (days 2, 6, 12). Scale bar, 100 µm. (H) Representative Masson's trichrome staining images of each group at different time points (days 2, 6, 12). Scale bar, 100 µm. Data are presented as means ± SDs.

We assessed the in vivo antibacterial performance of the hydrogel using a full‐thickness skin wound infection model (Figure 7A). Compared with the control and the QCO treatment groups, QCOR treatment significantly reduced the bacterial load (Figure 7B).

We further evaluated the wound‐healing performance of the hydrogels. To facilitate application in more challenging scenarios, wound infections were induced simultaneously using two clinically prevalent pathogens, MRSA and ESBLs‐*E. coli*. Treatments were administered on days 0, 2, 4, and 6 (Figure 7C). The wound images and corresponding wound‐area calculations on days 6 and 12 demonstrated that the QCOR hydrogel group exhibited significantly reduced wound areas compared with the control group at both time points, effectively promoting the healing of infected wounds in mice. On day 12, the wound‐healing rate of the QCOR hydrogel group was 99.4% (Figure 7D,E,F). Consistent with these findings, H&E and Masson's trichrome staining revealed that the QCOR hydrogel group exhibited a promising recovery trend, with complete epithelial regeneration and well‐aligned collagen fiber arrangement (Figure 7G,H). Statistical analysis further demonstrated that the QCOR group exhibited greater epithelial thickness and enhanced collagen deposition (Figure S8A,B). In summary, the QCOR hydrogel exhibited potent therapeutic efficacy in murine models.

## 3 Discussion

AMPs offer a potential alternative to antibiotics for treating drug‐resistant bacterial infections; however, their clinical application is constrained by their instability. Various chemical‐modification strategies have been developed to address this limitation [57], of which D‐amino acid substitution has emerged as one of the most effective and widely adopted approaches [58]. Nevertheless, such modifications compromise antimicrobial potency in some cases, and high‐throughput screening for optimal variants requires substantial resources and time. Recently, AI has been applied to the development and screening of AMPs through global and local searches [59, 60]. Limited by the lack of a D‐amino acid AMP dataset and challenges in modelling D‐peptide conformations, AI approaches for D‐peptide property prediction remain underexplored. Constructing a D‐amino acid AMP database could facilitate the prediction of antimicrobial activity following D‐amino acid substitution. Therefore, our study used AI to predict changes in AMP antimicrobial activity after D‐amino acid substitution without relying on structural information, thereby enabling efficient screening of D‐amino acid substituted AMPs.

On this basis, we conducted the following examinations to achieve the dual aims of high antibacterial potency and stability: (1) Construction of the first curated benchmark that systematically paired D‐substituted AMP sequences with quantitative antimicrobial activity, enabling rigorous and reproducible algorithmic evaluation; and (2) Development of ADAPT, a transparent, sequence‐based predictor that accurately estimated the dual impact of D‐substitutions on potency and proteolytic stability without relying on 3D structural models. EDSS was implemented as an efficient search strategy that navigated the exponential substitution landscape to identify useful variants with minimal computational overhead.

Our curated repository of D‐substituted AMP sequences and antimicrobial activities, annotated via positional one‐hot encoding, provided a functionally predictive baseline. To leverage prior knowledge of protease‐cleavage sites and the mirror–image conformational preferences of Damino acids, we developed a SMILES–diagram–based representation. Incorporating these structural priors into the feature set produced a marked improvement in prediction accuracy. Beyond prediction, we addressed a core challenge of designing: given a native AMP sequence of length *L*, each residue may be either L– or D–configured, resulting in a search space of size 2^*L*^ − 1 (e.g., 2^14^ approximately 1.6 × 10^4^ for *L* = 14). The scarcity of experimentally validated D‐substituted AMPs made standard generative models prone to overfitting and poor out‐of‐domain generalization. To overcome these limitations, we introduced an unsupervised domain adaptation fine‐tuning procedure to align the domain gap and performed an efficient combinatorial search over the 2^*L*^ − 1 substitution patterns. Experimental results highlighted the power of combining simple one‐hot sequence encoding, SMILES figures with protease‐cut‐site priors, and an efficient screening pipeline to identify D‐amino acid modified AMPs.

Conventional optimization of AMPs through D‐amino acid substitution remains empirically guided and inefficient, owing to the absence of generalizable design rules, with existing methods achieving limited success. We developed an AI‐based pipeline that substantially improved the success rate: 80% of AI‐designed variants derived from the two peptide templates exhibited elevated antimicrobial activity. Among the peptide candidates, dR2‐1 exhibited the greatest potential for clinical translation. Both in vitro and in vivo experiments confirmed the biological functions of the lead peptide. It displayed potent antimicrobial activity against a broad range of clinically isolated drug‐resistant pathogens, did not induce resistance in serial‐passage assays, showed low host toxicity, and demonstrated substantially improved stability relative to the template peptide. dR2‐1 acted primarily via membrane disruption, enabling rapid bactericidal action, and also exhibited potent antibiofilm activity. For a common clinical application of AMPs, we selected a skin infection wound model and used polysaccharide‐based hydrogels as a delivery vehicle, achieving dual functions of antibacterial efficacy and wound healing with good biocompatibility.

The lack of integrated 3D structural information represented a limitation of the current framework, as it could mask conformational effects of substitutions on activity. Future advances in the structural prediction for peptides containing D‐amino acids (for example, D‐flow, an all‐atom flow‐based framework) are expected to enhance model performance [61]. Integrating 3D structural information to reconcile stereochemical and physicochemical properties will be an important direction for model development. Moreover, expansion of experimental datasets for D‐form AMPs would facilitate multidimensional optimization of therapeutic candidates, including toxicity and immunogenicity. These advances will improve AI‐based AMP optimization transparency and enable engineering of next‐generation peptides with balanced properties (efficacy, stability, and safety), thereby closing the loop between computational design and molecular understanding.

## 4 Conclusion

In summary, this study established an open‐source database of D‐amino acid substituted AMPs and developed a machine‐learning model to predict the antimicrobial activity, thereby addressing a key gap in D‐AMP engineering by simultaneously satisfying the dual requirements of biological activity and stability. Wet‐lab assays showed that 80% of the predicted variants exhibited enhanced antimicrobial activity. Among these candidates, the lead peptide dR2‐1, which featured a strategically placed single‐site D‐residue, demonstrated greater potency, and stability under various conditions than the parent peptide, together with a broader therapeutic window. Mechanistic studies confirmed a membrane‐targeting antibacterial mode of action. In a skin‐infection murine model, dR2‐1 significantly reduced bacterial burden and accelerated wound healing when delivered using a biocompatible hydrogel‐based system. This optimization approach produced AMPs with improved clinical‐translation potential. The findings offer an innovative solution that addresses the persistent bottleneck in peptide optimization while contributing a strategy to combat rising antimicrobial resistance.

## 5 Experimental Section

### 5.1 Public Datasets Containing D‐Amino Acid Substituted AMPs

We constructed a dataset named QLAPD‐D, comprising D‐amino acid‐containing AMPs with all‐L counterparts. The dataset was constructed through a systematic process of literature mining and database curation, followed by filtering and pairwise construction.

1. Literature Mining and Initial Collection

We performed a comprehensive literature search using the PubMed database. The search strategy used the following Boolean query: (“antimicrobial peptide*” OR “AMP”) AND (“D‐amino acid” OR “D‐form” OR “diastereomer” OR “enantiomer”). Inclusion criteria for this stage were: (1) studies reporting novel or known AMPs incorporating at least one D‐amino acid substitution; (2) availability of the full‐length amino acid sequence; (3) reporting of quantitative antimicrobial activity against bacterial strains.

2. Database Integration and Curation

To ensure comprehensiveness, we cross‐referenced the collected peptides with the DBAASP (Database of Antimicrobial Activity and Structure of Peptides) database. We queried DBAASP using the “All Modifications” option within the “Unusual Amino Acid” field and subsequently filtered the results to isolate peptides featuring D‐amino acid modifications. Then we merged the results with our literature‐derived collection, removing duplicates based on sequence and modification identity.

3. Data Filtering

The combined pool of peptides was subjected to a two‐step filtering process: Primary Filter: We excluded peptides with non‐standard backbone structures (cyclopeptides and stapled peptides) and other non‐canonical amino acids to focus on linear AMPs with D‐amino acid modifications. Secondary Filter: We retained only those D‐amino acid‐containing peptides for which the sequence and activity data of the exact, corresponding all‐L‐amino acid counterpart were also available in the source literature or database. This was crucial for ensuring a valid comparative analysis.

4. Pairwise Dataset Construction

From the final list of 815 qualified AMPs, we constructed a pairwise training dataset. For a given parent all‐L sequence and its modified variant(s), every possible pairwise combination between the all‐L peptide and its D‐substituted version(s) was formed. This procedure resulted in a total of 2,837 unique sequence‐activity pairs for model training.

These paired data form two datasets for separate tasks:

1. Classification task. Samples with a reduced geometric mean MIC to their parent sample (enhanced activity) are labeled positive, while those with an unchanged or increased geometric mean MIC (diminished activity) are labeled negative.
2. Regression task. Each label is defined as
$$y=\log_{2}\left({\mathrm{MIC}}_{\mathrm{mut}}/{\mathrm{MIC}}_{\mathrm{parent}}\right)$$
  ensuring a symmetric numerical range. When a peptide has multiple MIC measurements against different microbes, its geometric mean is taken as the single MIC value.

### 5.2 Wetlab Validated Dataset for Evaluation

We systematically developed a fully wet‐lab validated dataset to provide a high‐quality and reliable D‐amino acid AMP research benchmark. Unlike previous studies that rely on heterogeneous or predicted data, using R2 as a template, we employed strategies such as random substitution and prior knowledge‐guided restricted sites to synthesize and construct the R2 wet‐lab‐validated dataset for model testing: MICs for all peptides were experimentally determined against drug‐resistant bacterial strains. This comprehensive and experimentally validated dataset provides a robust benchmark for evaluating D‐amino acid substitution strategies.

### 5.3 Detailed Design of ADAPT Model

The structure of ADAPT model is shown in Figure 2D. In the left branch, the sequence encoder employed a bidirectional Mamba to capture long‐range dependencies in the amino acid sequence. SMILE images were constructed to capture covalent interactions, complementing the sequence encoder, with D‐substituted residues visually emphasized via blue bonds overlay. This approach increased the salience of chiral inversions to the downstream image encoder. Additionally, protease cleavage hot‐spot bonds were overlaid as red, enabling the model to anticipate stability trade‐offs introduced by D‐substitutions. These cleavage rules were inferred from experimentally mapped protease motifs [24] with details in Table S1. The right branch leverages a ImageNet‐pretrained ResNet‐18 to extract structural information from above mentioned 2D SMILE rendering of the peptide. The fusion of features from the Siamese network was achieved via a simple concatenation operation.

To train the model for classification, a cross‐entropy loss function is applied, defined as:

(1) $$L=-\frac{1}{N}\sum_{i=1}^{N}\left[y_{i}\log\left({\hat{y}}_{i}\right)+\left(1-y_{i}\right)\log\left(1-{\hat{y}}_{i}\right)\right]$$

where *y*_*i*_ is the ground truth label indicating whether the D‐substituted peptide is more antimicrobial active, and ${\hat{y}}_{i}$ is the predicted probability. This loss ensures that the model effectively learns to distinguish stability levels between L‐ and D‐substituted peptides.

To train the model for regression, a Mean Squared Error (MSE) loss function is applied, defined as:

(2) $$L_{\mathrm{MSE}}=\frac{1}{N}\sum_{i=1}^{N}{\left(s_{i}-{\hat{s}}_{i}\right)}^{2}$$

where *s*_*i*_ is the ground truth log_2_ MIC ratio of the *i*‐th peptide pair and ${\hat{s}}_{i}$ is the model's predicted score. By penalizing the squared difference between true and predicted values, this loss encourages the model to make accurate continuous predictions and heavily discourages large errors.

We address performance degradation due to source–target domain shift by formulating training as unsupervised domain adaptation (UDA) with a mean‐teacher framework [19, 28, 29, 30, 31, 32, 33]. The labeled QLAPD‐D dataset served as the source domain, and the combinatorial EDSS space of candidate L→D mutants for a peptide of length *n* defines the target domain:

(3) T={all L→D substitution subsets},

(4) $$|T|\;=\;2^{n}-1$$

A student network was optimized on source data with the supervised ADAPT‐CLS (cross‐entropy) and ADAPT‐REG (mean‐squared error) objectives, while a teacher network provided stable targets on unlabeled target data via consistency regularization under stochastic augmentations. The teacher parameters were updated as an exponential moving average (EMA) of the student parameters:

(5) $$\theta_{\text{tea}}\;\leftarrow \;\alpha \;\theta_{\text{tea}}\;+\;\left(1-\alpha \right)\;\theta_{\text{stu}}$$

which propagates information from labeled to unlabeled domains and stabilizes training. The total objective combined supervised and consistency losses:

(6) $$L\;=\;L_{\text{sup}}\;+\;\lambda \;L_{\text{cons}}$$

where

(7) $$L_{\text{sup}}\;=\;L_{\text{CE}}\;+\;L_{\text{MSE}}$$

(8) $$L_{\text{cons}}\;=\;D\;\left(f_{\text{stu}}\left(x^{T}\right),\;f_{\text{tea}}\left({\tilde{x}}^{T}\right)\right)$$

here, $x^{T}$ and ${\tilde{x}}^{T}$ denote stochastically augmented target‐domain inputs, *f*_stu_ and *f*_tea_ are the student and teacher predictors, *D* is a divergence (e.g., squared error for regression and KL divergence for classification), and λ controls the strength of the unsupervised signal. This UDA design leverages the full target distribution without additional labels, improving generalization across the expansive mutant space.

### 5.4 Model Implementation and Evaluation

All methods were optimized using AdamW (weight‐decay of 10^−2^) at learning rate 10^−3^. Early stopping was used to ensure identical optimization trajectories. Models were trained for 35 epochs; final metrics equal the mean of the last‐epoch scores across five stratified folds. The classification was assessed using AUROC and Average Precision. We implemented a stratified fivefold cross‐validation protocol to obtain an unbiased estimate of generalization performance. The external dataset was partitioned into five mutually exclusive folds; at each iteration, fourfolds were used for training and the remaining fold for validation.

Experiments were executed on Ubuntu 22.04 with an Intel Core i9‐14900K CPU, 192 GB RAM, and two NVIDIA GeForce RTX 4090 D GPUs (24 GB VRAM each). Storage was provided by a 4 TB Samsung 990 PRO SSD. The software stack consisted of Python 3.10, PyTorch 2.2.0 [62], Biopython, 1.78 [63] and TorchMetrics 1.4.0 [64], RDKit 2024.3.5. GPU drivers were NVIDIA 550.144.03 with CUDA 12.4.

### 5.5 Reagents and Bacterial Strains

Müller‐Hinton broth (MHB) was purchased from Hopebio (China). Polymyxin B, LPS, ABTS free radical scavenging assay kits, SDS, ROS assay kits and H&E staining kits were obtained from Solarbio (China). NPN was purchased from Aladdin. ONPG and 4‐(2‐hydroxyethyl)piperazine‐1‐ethanesulfonic acid (HEPES) were obtained from Thermo Fisher (USA). Cell Counting Kit‐8 (CCK‐8) was purchased from Biosharp (China). Gentamycin sulfate, vancomycin, QCS (degree of substitution: 46.5%, as shown in Figure S9), ethylene glycol and glutaraldehyde were purchased from Macklin (China). Dextran (MW: 64,000–76,000) was obtained from Yuan Ye (China). Sodium hydroxide and hydroxylamine hydrochloride were provided by Sinopharm (China). Sodium periodate was supplied by Sigma (USA). BeyoBCA Peptide Quantitation Assay Kit (Colorimetric) was purchased from Beyotime (China).

Standard strains were purchased from China Center of Industrial Culture Collection (CICC) and National Center for Medical Culture Collections (CMCC). Clinical isolates were obtained from the clinical laboratory of Qilu Hospital of Shandong University. The detailed information of bacterial strains was shown in Table S8.

### 5.6 Characterization of AMP

#### 5.6.1 Antibacterial Activity

All peptide variants were synthesized using standard solid‐phase synthesis by GenScript (China) and confirmed by MS and HPLC assay (Figures S10 and S11). The MIC was determined using the microbroth dilution method, following Clinical and Laboratory Standards Institute (CLSI) guidelines. MHB was used to prepare two fold serial dilutions of peptides at final concentrations ranging from 0.5 to 256 µg mL^−1^. Each dilution was mixed with 50 µL of bacterial suspension [1× 10^6^ colony forming units (CFU)/mL] in the logarithmic growth phase in a 96‐well plate and incubated at 37°C for 18 h. MHB medium without bacteria served as the negative control, while bacterial suspension without peptides served as the positive control. All experiments were performed with three independent replicates.

#### 5.6.2 CD Spectroscopy

CD spectra of the peptides were measured at 25°C using a JASCO‐1500 spectropolarimeter (JASCO, Japan) equipped with a rectangular quartz cell (0.2 cm path length). The spectra were recorded from 190 to 260 nm at a scanning speed of 20 nm min^−1^, and the data were expressed as mean residue ellipticity. Measurements were performed under different environmental conditions: 10 mM PBS, 50% TFE and 30 mM SDS, with a final peptide concentration of 150 µM. The mean residue ellipticity was calculated using the following formula:

θ_M_ = (θ_obs_ × 1,000) / (c × l × n), where θ_obs_ is the observed ellipticity (in millidegrees) after buffer correction, c is the peptide concentration (in mM), l is the path length (in mm), and n is the number of amino acids in the peptide.

#### 5.6.3 Hemolytic Activity

The hemolytic activity of the peptides was evaluated using human red blood cells (RBCs). The RBCs were diluted to 8% (v/v) with sterile PBS. Then 100 µL of serially diluted peptide solution was mixed with 50 µL of the diluted RBC suspension in a 96‐well plate and incubated at 37°C for 1 h. After incubation, the samples were centrifuged at 1,000 × *g* for 5 min, and the supernatant was transferred to a flat‐bottom 96‐well plate. The absorbance was measured at 570 nm using a microplate reader (BioTek, USA). RBCs treated with PBS served as the negative control (OD_0_), while RBCs treated with 1% Triton X‐100 (v/v) served as the positive control (OD_100_). The percentage of hemolysis was calculated using the following formula:

Hemolysis (%) = [(OD_sample_−OD_0_) / (OD_100_−OD_0_)] × 100%

#### 5.6.4 Cytotoxicity

The cytotoxicity of the peptides was evaluated using the CCK‐8 assay to assess their effects on cell proliferation. 293T cells were seeded in a 96‐well plate (8,000 cells per well) and cultured in a 37°C, 5% CO_2_ incubator for 24 h. Subsequently, the cells were treated with varying concentrations of peptides, while control cells were treated with peptide‐free medium, followed by another 24 h of incubation. Then 10 µL of CCK‐8 reagent was added to each well, and the cells were further incubated for about 2 h. Absorbance was measured at 450 nm using a microplate reader (medium with CCK‐8 as blank). Cell viability was calculated using the following formula:

Cell Viability (%) = [(OD_sample_−OD_blank_) / (OD_control_−OD_blank_)] × 100%

#### 5.6.5 Salt, Serum, pH, and Protease Stability

The antimicrobial activity of the peptides under salt and serum conditions was tested in MHB medium supplemented with different components. The tested media included: MHB with 150 mM NaCl, 4.5 mM KCl, 2 mM CaCl_2_, 12.5% FBS or 25% FBS. For pH stability assay, peptide was incubated in MHB with pH = 5 or 9 for 1 h. To assess the resistance of peptides to proteolytic degradation, the peptide solutions were mixed with different concentrations of chymotrypsin solutions and incubated at 37°C for 1 h, followed by heat inactivation at 65°C for 15 min. The MIC was determined against *S. aureus* ATCC25923 and *E. coli* ATCC25922 following the procedure described in the antibacterial activity assay.

#### 5.6.6 Time‐Kill Assay

Bacterial suspensions of *S. aureus* ATCC25923, *P. aeruginosa* PAO1 and *A. baumanii* ATCC19606 in logarithmic growth phase were adjusted to 10^6^ CFU/mL. Then, 5 mL of the bacterial suspension was mixed with varying concentrations of AMP or antibiotics and incubated at 37°C. At designated time points (0, 10 min, 30 min, 1, 1.5, 2, 3, 4, and 6 h), samples were collected, serially diluted, and plated on agar plates. After incubation at 37°C for 24h, bacterial colonies were counted.

#### 5.6.7 Resistance Development Assay

Bacterial resistance development to AMPs and antibiotics was evaluated through serial sub‐MIC exposure. *S. aureus* CMCC26003 was treated with dR2‐1 and gentamycin. The MIC values of dR2‐1 and gentamycin were first determined. The bacteria were then cultured in MHB medium containing 1/2× MIC of the test agents. After treatment, the bacterial suspension was diluted to 1× 10^6^ CFU/mL for the next round of MIC determination. This process was repeated for 20 consecutive passages to monitor resistance trends.

### 5.7 Antimicrobial Mechanism Studies

#### 5.7.1 SEM Analysis

*S. aureus* ATCC25923 and *E. coli* ATCC25922 were cultured to the logarithmic phase, adjusted to OD_600nm_ = 1.0, and centrifuged at 3,000 rpm for 10 min. The pellets were washed with PBS and resuspended. Then bacterial suspension was mixed with an equal volume of AMP solution (4× MIC) and incubated at 37°C for 2 h. The mixture was centrifuged, and the pellet was fixed with 2.5% glutaraldehyde at 4°C overnight. Samples were washed with PBS and dehydrated in a graded ethanol series for 10 min each. After drying, samples were sputter‐coated with gold and imaged using a JSM‐6700F SEM (JEOL, Japan) to observe morphological damage.

#### 5.7.2 Outer Membrane Permeability Assay

*E*. *coli* ATCC25922 cultured to logarithmic phase was adjusted to OD_600nm_ = 0.1. After centrifugation (5,000 × *g*, 5 min), the pellet was washed three times with 5 mM HEPES buffer containing 5 mM glucose and resuspended. For the assay, 50 µL of AMP solutions at varying concentrations was mixed with 100 µL bacterial suspension, followed by addition of 50 µL NPN (40 µM) under dark conditions. Fluorescence intensity was measured every 3 min for 30 min using a microplate reader (excitation λ = 350 nm, emission λ = 420 nm), with PBS serving as the negative control.

#### 5.7.3 Cytoplasmic Membrane Permeability Assay

*E. coli* ATCC25922 was cultured in MHB medium supplemented with 2% lactose until the logarithmic growth phase was reached. The bacterial suspension was adjusted to OD_600nm_ = 0.1 and centrifuged at 5,000× *g* for 5 min, then the pellet was washed three times with PBS and resuspended. Subsequently, 100 µL of the bacterial suspension was mixed with 50 µL of AMP solutions at varying concentrations and 50 µL of 6 mM ONPG solution. The absorbance at 420 nm was measured every 5 min for 90 min using a microplate reader. PBS was used as the negative control.

#### 5.7.4 Cytoplasmic Leakage Assay

*E. coli* ATCC25922 in logarithmic phase (adjusted to OD_600nm_ = 0.1) was centrifuged (1,000× *g*, 10 min) and washed three times with PBS. After 1:1 mixing with peptide solutions, the mixtures were incubated at 37°C. Samples (1 mL) were collected at 1, 3, 5, 7, and 24 h and centrifuged (12,000 rpm, 2 min). Supernatants were collected and analyzed for DNA/RNA/protein content using Nanodrop (Thermo Fisher, USA), with PBS as the negative control.

#### 5.7.5 LPS Competitive Inhibition Assay

Peptide‐LPS interaction was evaluated by antimicrobial activity post‐LPS treatment. Polymyxin B served as positive control. After pre‐incubation [25 µL 4× MIC peptide solution and 25 µL serially diluted LPS (1‐1,024 µg mL^−1^)] at 37°C for 1 h, 50 µL *E. coli* ATCC25922 suspension (1×10^6^ CFU/mL) was added and incubated for 18 h. Then OD_600nm_ was measured.

#### 5.7.6 ROS Generation Assay

*S. aureus* ATCC25923 and *E. coli* ATCC25922 in logarithmic phase was adjusted to OD_600nm_ = 0.3. The bacterial suspension was co‐incubated with 10 µM 2',7'‐Dichlorodihydrofluorescein diacetate (DCFH‐DA) at 37°C for 30 min, then was washed by PBS for three times. Then, 190 µL of the mixture was co‐incubated with 10 µL of AMPs at different concentrations for 15 min. Fluorescence intensity was measured using a microplate reader (excitation λ = 488 nm, emission λ = 525 nm).

#### 5.7.7 Antibiofilm Activity Assay

For the biofilm inhibition assay, 50 µL of peptide solution (final concentrations: 2×, 1×, 1/2×, 1/4× MIC) was mixed with 50 µL of *S. aureus* CMCC26003 suspension (1×10^6^ CFU/mL in TSB medium with 1% glucose). TSBG medium (1% glucose) without peptides was used as positive control. After incubation for 20–24 h at 37°C, the medium was discarded, and wells were washed with PBS. The biofilm was fixed with methanol (15 min), air‐dried, and stained with 0.1% (w/v) crystal violet (15 min). Then excess dye was removed, and the biofilm was dissolved in 30% (v/v) acetic acid. Absorbance was measured at 595 nm (30% acetic acid as blank). For biofilm eradication, after 24 h of bacterial incubation, peptide solutions (final concentrations: 2×, 1×, 1/2×, and 1/4× MIC) were added and incubated for an additional 24 h. The remaining steps followed the same protocol as the biofilm inhibition assay.

### 5.8 Mouse Skin Infection Model

Female BALB/c mice (8 weeks) were purchased from Beijing Vital River Laboratory Animal Technology Co., Ltd. and used to establish a skin infection model for evaluating the antibacterial efficacy of AMP. The dorsal hair of the mice was shaved, and a full‐thickness wound (5 × 5 mm) was created on the back. The wound was inoculated with 5 µL of *S. aureus* ATCC25923 suspension (1 × 10^8^ CFU/mL). In the single‐dose treatment group, PBS or AMP was administered at 4 h after infection. In the multi‐dose group, administrations were performed at 4 and 12 h post‐infection. Skin samples were collected 24 h post‐infection for quantitative analysis of bacterial load.

### 5.9 In Vivo Safety Evaluation

Female mice (8 weeks, n = 8) were administered intraperitoneally with 200 µL of AMP solutions at varying doses (20, 40, and 60 mg kg^−1^). PBS served as negative control. Survival rates were monitored continuously for 7 days, and the LD_50_ was calculated based on the results. At the experimental endpoint, blood samples were collected for biochemical analysis to evaluate liver and renal functions. Additionally, five major organs (heart, liver, spleen, lungs, and kidneys) were harvested, fixed, and processed into paraffin‐embedded sections for H&E staining to assess potential organ damage.

### 5.10 Design, Synthesis, and Characterization of Hydrogels

#### 5.10.1 Synthesis and Characterization of ODEX

The synthesis of ODEX was performed according to previously reported methods [65, 66, 67]. Briefly, 5 g of DEX was dissolved in 200 mL of deionized water, followed by the addition of 5 g of NaIO_4_. The reaction was allowed to proceed under dark conditions with stirring for 2.5 h. Subsequently, 5 mL of ethylene glycol was added to terminate the reaction, followed by an additional 30 min of stirring. The product was then dialyzed (MWCO: 3,500Da) for 5 days, lyophilized, and stored at 4°C for further use. The degree of oxidation was determined using the hydroxylamine hydrochloride‐methyl orange titration method.

#### 5.10.2 Hydrogel Preparation

For hydrogel preparation, 0.4 g QCS was dissolved in deionized water (10 mL) to form precursor solution (4% w/v). ODEX (5% w/v) was prepared similarly. The QCO hydrogel was formed by mixing equal volumes of QCS and ODEX solutions. To prepare drug‐loaded hydrogels, dR2‐1 was completely dissolved in QCS solution to form QCR precursor (the final concentration of dR2‐1 was 4‰w/v), which was then mixed with ODEX solution at 1:1 ratio to obtain the QCOR hydrogel. The formation of Schiff base bonds and chemical group modifications in hydrogels were verified by Nicolet IS5/IS10 FTIR (Thermo Fisher, USA) with spectral scanning from 4,000 to 400 cm^−1^.

#### 5.10.3 Rheological Properties of Hydrogels

The rheological properties of the hydrogels were measured using a DHR‐2 rheometer (TA, USA). During testing, the hydrogel samples were placed between parallel plate fixtures with a gap set at 2 mm. At room temperature, frequency sweeps (0.1–10 Hz) and strain sweeps (0.1%–10%) were performed to evaluate the variations in storage modulus (G') and loss modulus (G'’), thereby characterizing the rheological behavior of the hydrogels.

#### 5.10.4 Self‐Healing, Injectable, and Adhesive Properties

The self‐healing capability was examined by cutting the hydrogel discs into two halves and bringing the cut surfaces into contact for 5 min before evaluating the structural integrity. The injectability of hydrogels was assessed by extruding QCOR hydrogel through syringe needles to form the letters SDU. Adhesive properties were tested by applying hydrogels onto finger and assessing stretchability and tissue adhesion performance.

#### 5.10.5 Swelling and Degradation Properties

Hydrogel samples (500 µL) were lyophilized and placed in 1.5 mL EP tubes to record the initial weight (W_0_). Each tube was then filled with 1 mL PBS and incubated at 37°C. At predetermined time intervals, the samples were carefully removed, surface moisture was absorbed with filter paper, and the weight (W_n_) was recorded before reimmersion in fresh PBS. Upon reaching swelling equilibrium, the weight was recorded as W_1_. The swelling ratio (%) was calculated as [(W_n_–W_0_)/W_0_]×100, while the degradation ratio (%) was determined as [(W_1_ –W_n_)/W_1_]×100.

#### 5.10.6 Morphological Characterization by SEM

The microstructural features of QCO and QCOR hydrogels were examined using a HITACHI SU8010 field emission SEM (HITACHI, Japan).

#### 5.10.7 In Vitro Drug Release Assay

The drug release kinetics was evaluated by immersing hydrogels in sterile PBS solution with pH = 5.0 or 7.4 (0.1 g mL^−1^) at 37°C. At predetermined time points, aliquots were withdrawn and analyzed using the BeyoBCA Peptide Quantitation Assay Kit (Colorimetric) according to the manufacturer's protocol. The absorbance at 480 nm was measured using a microplate reader.

#### 5.10.8 Hemolytic Activity of Hydrogels

Hemolytic activity was assessed using 8% (v/v) RBC suspension. 0.1 mL hydrogel was mixed with 1 mL RBC suspension in 1.5 mL EP tubes and incubated at 37°C for 1 h. After centrifugation (1,000 × *g*, 5 min), 150 µL supernatant was transferred to 96‐well plates for absorbance measurement at 570nm. 0.2% Triton X‐100 (v/v, OD_100_) and PBS (OD_0_) served as positive and negative controls, respectively.

Hemolysis rate was calculated as: Hemolysis (%) = [(OD_sample_–OD_0_)/(OD_100_–OD_0_)]×100.

#### 5.10.9 Hydrogel Cytocompatibility Assessment

The cytocompatibility of the hydrogels was evaluated using the CCK‐8 assay. The hydrogels (100 mg) were immersed in 1 mL of complete Dulbecco's Modified Eagle Medium (DMEM) (DMEM containing 10% FBS, 100 U/mL penicillin and 0.1 mg mL^−1^ streptomycin) and incubated at 37°C for 24 h to obtain the extraction solutions. 293T cells were seeded in a 96‐well plate at a density of 8,000 cells/well and cultured in a humidified incubator at 37°C with 5% CO_2_ for 24 h. After removing the culture medium, the hydrogel extraction solutions were added, followed by another 24 h of incubation. Cell viability was then assessed using the CCK‐8 reagent, following the procedure described in the peptide cytotoxicity test.

#### 5.10.10 Evaluation of Hydrogel Antioxidant Capacity In Vitro

ABTS radical scavenging activity was measured using ABTS free radical scavenging assay kit according to manufacturer's instructions. Absorbance at 405 nm was measured by microplate reader. Clearance rate was calculated as:

D% = [(A_0_‐(A_sample_–A_n_))/A_0_]×100%, where A_0_ was blank control, A_sample_ was test sample, and A_n_ was negative control.

### 5.11 Evaluation of Antibacterial Properties of Hydrogels In Vitro

#### 5.11.1 Colony Forming Unit Assay

Bacterial strains including *E. coli* ATCC25922 and *S. aureus* ATCC25923 were cultured to logarithmic phase and adjusted to 10^7^ CFU/mL. In 24‐well plates, 100 µL hydrogel was placed at the bottom, followed by addition of 1 mL bacterial suspension. After 8 h incubation at 37°C, bacterial suspensions were serially diluted, plated, and incubated for 24 h at 37°C for colony counting and photographing. Bacteria‐free culture medium served as blank control, while bacterial suspension served as negative control.

#### 5.11.2 Inhibition Zone Assay

Bacteria in the logarithmic phase were adjusted to OD_600nm_ = 0.1. *S. aureus* ATCC25923 or *E. coli* ATCC25922 suspension (100 µL) was evenly spread on agar plates. Hydrogel discs (1 cm in diameter, 2 mm thickness) were placed centrally on the agar and incubated at 37°C for 24 h to observe inhibition zone formation.

### 5.12 Evaluation of Antibacterial Properties of Hydrogels In Vivo

Female BALB/c mice (8 weeks) were used in this study and divided into three groups, with six mice in each group. Prior to surgery, mice were anesthetized by intraperitoneal injection of 1% sodium pentobarbital solution (50 mg kg^−1^). Folowing dorsal hair removal, a standardized circular full‐thickness wound (8 × 8 mm) was created on the midline back region and immediately infected with 5 µL of MRSA 103 (10^8^ CFU/mL). After one hour of bacterial inoculation, wounds were treated with 50 µL of either QCO hydrogel, QCOR hydrogel, or sterile PBS (control), followed by cover with 3M Tegaderm dressing. At 24 h post‐treatment, mice were euthanized for tissue collection. Excised wound tissues were homogenized and subjected to serial dilution plating on agar for quantitative bacterial counting (CFU/biopsy) after 24 h incubation at 37°C.

### 5.13 Wound Healing Assessment

Female BALB/c mice (8 weeks) were randomly divided into four groups: PBS, QCO, QCOR, and dR2‐1 (n = 6). Under anesthesia, dorsal hair was removed and a standardized 10 × 10 mm circular full‐thickness skin wound was created. The wounds were then inoculated with 5 µL of MRSA 103 (10^8^ CFU/mL) and 5 µL of extended‐spectrum β‐lactamase‐producing *E. coli* 208 (ESBLs‐*E. coli*) (10^8^ CFU/mL) bacterial suspensions. At 24 h post‐infection, wounds were treated with 100 µL PBS, QCO, QCOR, or dR2‐1, with subsequent treatments administered on days 2, 4, 6. Tissue samples were collected on days 3, 7, and 12 for histopathological analysis. Wound progression was documented by photographic imaging. Wound area measurements, epithelial thickness, and collagen deposition were quantified using ImageJ software. Excised wound tissues were fixed, paraffin‐embedded, and sectioned for H&E and Masson's trichrome staining.

### 5.14 Ethical Approval

All experimental protocols were approved by the Animal Ethics Committee of Qilu Hospital, Shandong University (Approval No. DWLL‐202500148), with strict adherence to institutional guidelines for humane endpoint monitoring and postoperative care.

### 5.15 Statistical Analysis

Data are presented as mean ± standard deviation (SD). Statistical significance between two groups was determined by t‐test or Mann–Whitney U‐test, whereas multiple group comparison was determined by one‐way ANOVA with Dunnett's multiple comparison test in GraphPad Prism 9.5.1.

## Author Contributions

Conceptualization: Y.L., H.W., Y.Z., Q.K., and H.G.; Methodology: Y.Z., Q.K., and H.G.; Investigation: Y.Z., Q.K., H.G., L.L., B.W., P.W., J.F., X.L., Y.W., J.Z., Y.Y., X.Y., and X.Z.; Visualization: Y.Z., Q.K., H.G., and B.W.; Funding acquisition: Y.L.; Project administration: Y.L., and L.L.; Supervision: Y.L., X.Z., X.Y., and Y.Y.; Writing – original draft: Y.Z., Q.K., and H.G.; Writing – review & editing: Y.L., H.W., Y.Z., Q.K., and H.G.

## Conflicts of Interest

The authors declare no conflicts of interest.

## Supporting information


**Supporting File**: advs73833‐sup‐0001‐SuppMat.docx.

## Data Availability

The data related to this work are available in the supplementary data files. The source codes and trained models are made publicly available at https://github.com/EricwanAR/DAminoMuta.
