# Supplementary material for: AI‐Based D‐Amino Acid Substitution for Optimizing Antimicrobial Peptides to Treat Multidrug‐Resistant Bacterial Infection
Source: Adv Sci (Weinh). 2026 Jan 14;13(10):e18522. doi: 10.1002/advs.202518522 (PMC12915103; doi:10.1002/advs.202518522)
Supplement: Supplementary file 1 — Supporting File: advs73833‐sup‐0001‐SuppMat.docx. [file ADVS-13-e18522-s001.docx]

Supporting Information

AI-based D-amino acid substitution for optimizing antimicrobial peptides to treat multidrug-resistant bacterial infection

*Yinuo Zhao^1,†^, Qingzhou Kong^1,†^, Haifan Gong^1,2,†^, Lixiang Li^1,3,4^, Jialu Fu^1^, Boyao Wan^1^, Peizhu Wang^1^, Xiaojuan Li^1^, Yue Wang^1^, Jinghui Zhang^1^, Yanbo Yu^1,3,4^, Xiaoyun Yang^1,3,4^, Xiuli Zuo^1,3,4^, Haina Wang^5,*^, Yanqing Li^1,3,4,*^*

*^*^Corresponding author(s). E-mail(s): whn2013@sdu.edu.cn (H.W.); liyanqing@sdu.edu.cn (Y.L.).*

*
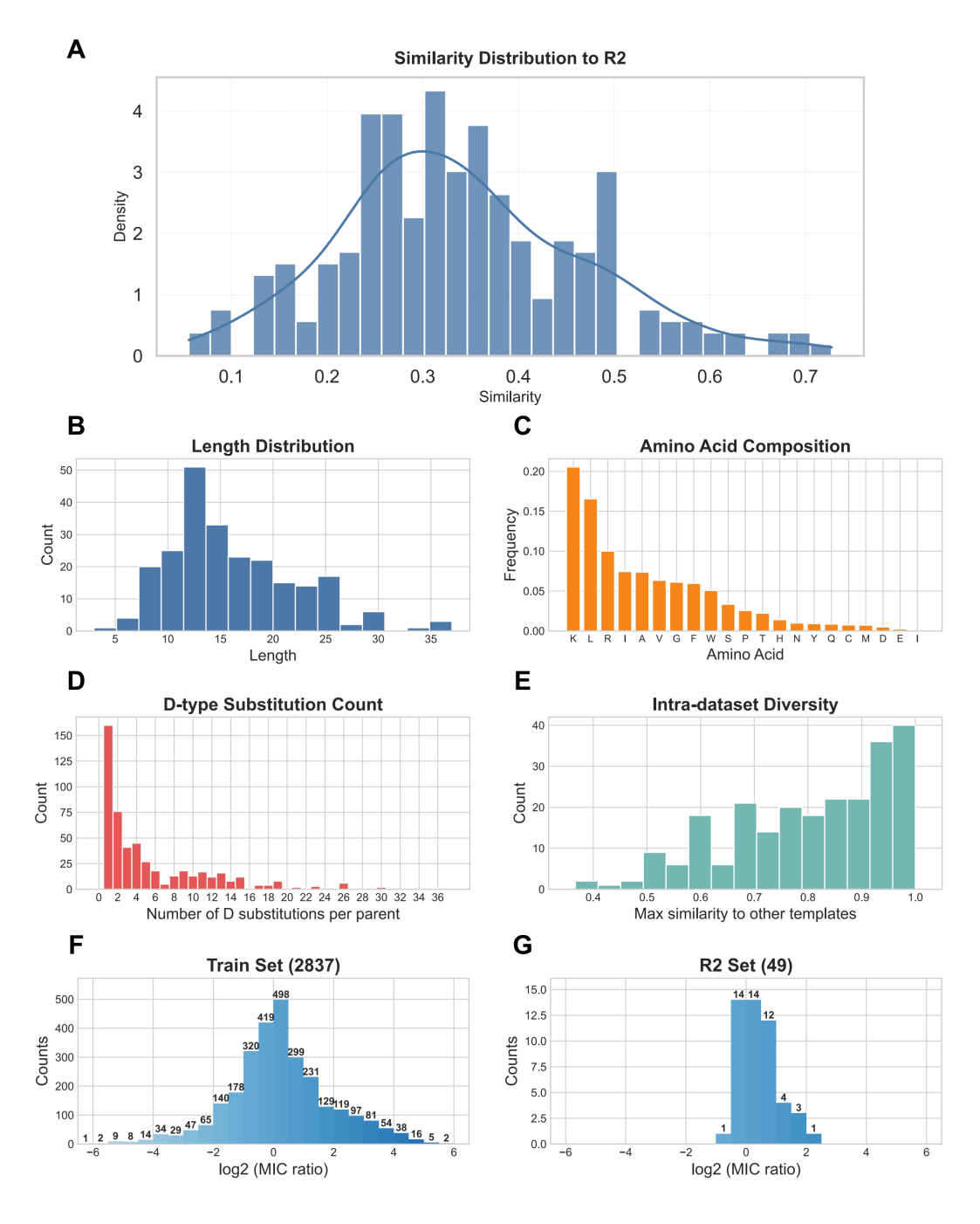
*

**Figure S1. Detailed statistical analysis of the QLAPD-D dataset.**

(**A)** Similarity distribution between dataset templates and the R2 antimicrobial peptide reference; the highest similarity (0.7273) is observed for the template KLWKKWKKWLK is still quite low, indicating the out-of-domain generalization issue.

(**B)**Length distribution of L-type template peptides in the dataset.

(**C)**Amino acid composition frequencies across template sequences, sorted in descending order.

(**D)** Distribution of D-type amino acid substitutions per variant.

(**E)** Intra-dataset diversity represented by the maximum pairwise similarity of each template to all other templates.

(**F)** MIC ratio (D-amino acid substituted MIC/original MIC, where the MIC indicates the geometric average of MIC values from all bacteria species) distribution of train set, containing 2837 sample pairs.

(**G)** MIC ratio distribution of R2 test set, containing 49 sample pairs.


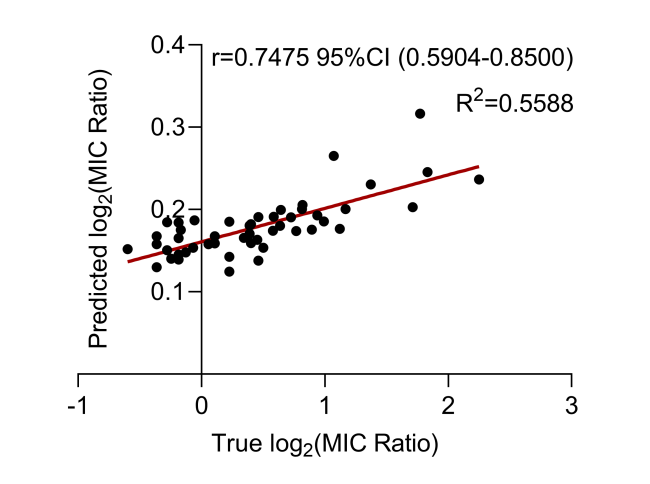


**Figure S2.** **Correlation between predicted activity scores and experimental MIC ratios.**

Scatter plot showing the relationship between computational predictions and experimentally determined MIC ratios (geometric mean MIC of substituted peptide/original peptide). Pearson *r* = 0.7475 (95% CI: 0.5904-0.8500).


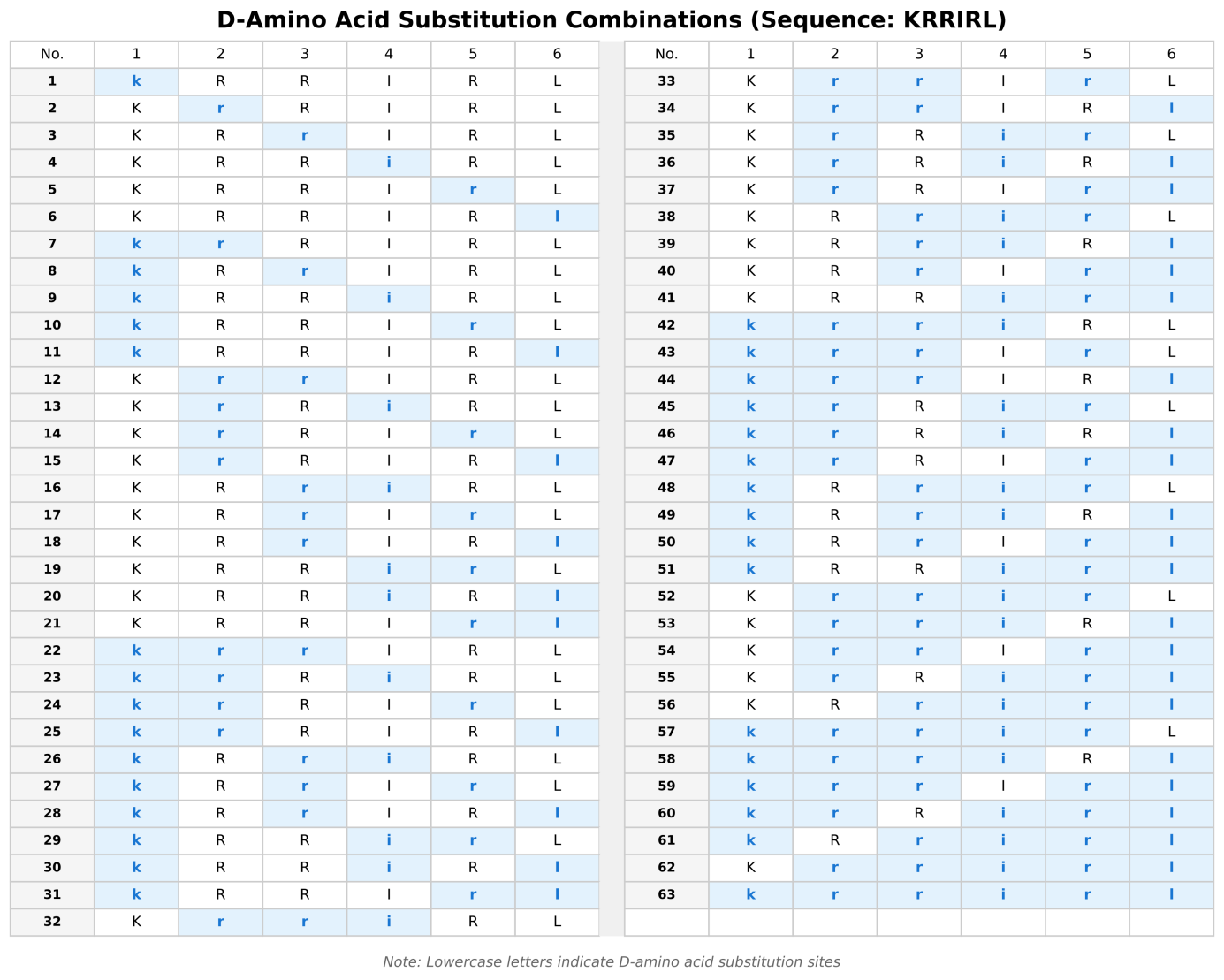


**Figure S3.** Example of D-amino substitution space of a peptide of length 6, combination space = 2^n^-1, where n indicates the sequence length and equals to 6 in this case.


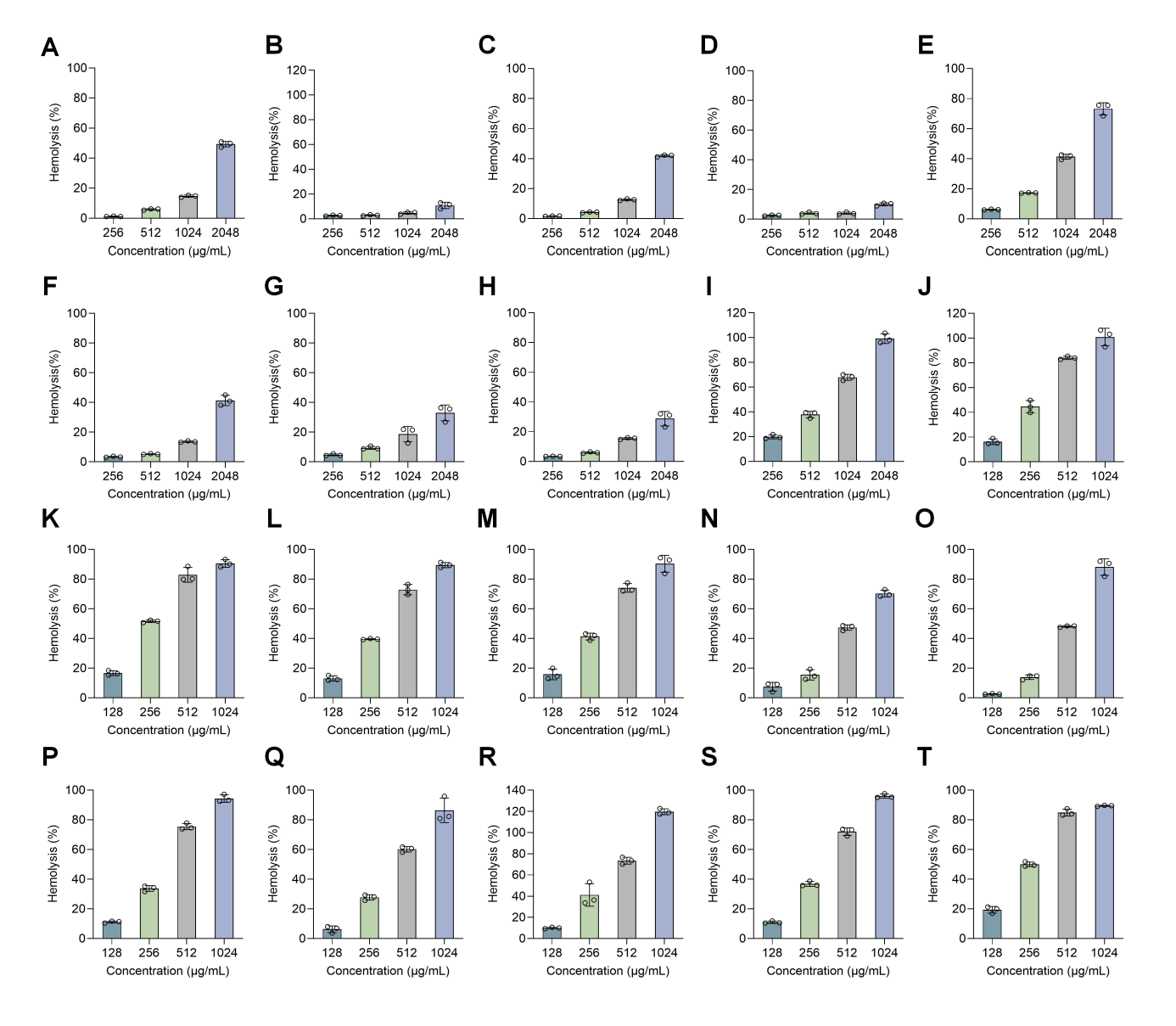


**Figure S4. Hemolytic activity of peptides.**

(**A** to **T**) Hemolytic activity of dR2-2 to dR2-10 (A to I), indolicidin (J) and dIndo-1 to dIndo-10 (K to T) on red blood cells, n=3.


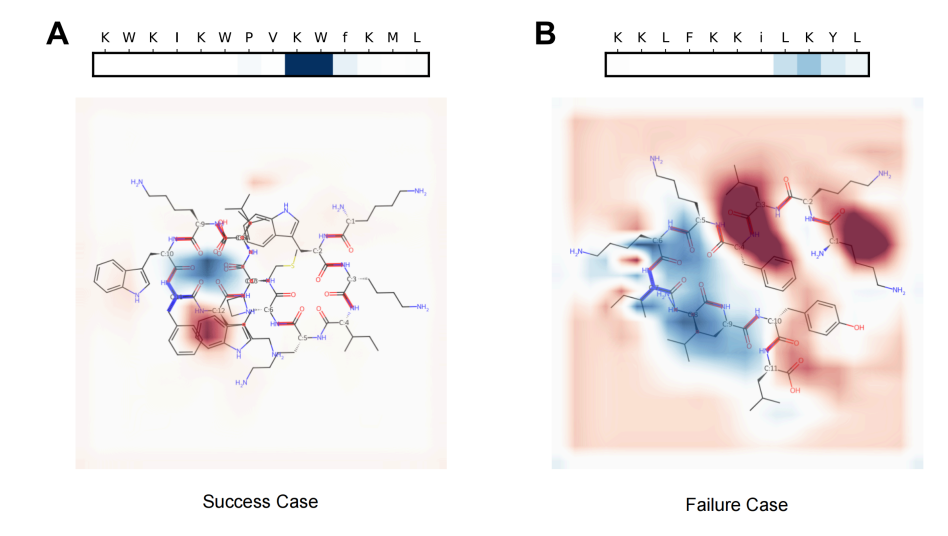


**Figure S5. Grad-CAM differential analysis of D-amino acid substitution effects on antimicrobial peptide activity.**

**(A)** Success case: Accurate prediction of activity change for peptide KWKIKWPVKWFKML with D-amino acid substitution at position 10 (lowercase f indicates D-Phe). The differential heatmap (D-substituted minus all-L parent peptide) reveals the model correctly identifies the local conformational perturbations induced by chirality inversion. Red regions indicate increased attention/positive contribution to activity enhancement, while blue regions suggest decreased attention or negative impact. The three bonds highlighted in blue represent the chiral center (C_α_) of the D-amino acid. Focused attention around the substitution site and adjacent residues demonstrates the model's ability to capture stereochemistry-dependent structural changes.

**(B)** Failure case: Misclassification for peptide KKLFFKILKYL with D-Ile substitution at position 7. The differential attention map shows widespread, incoherent activation patterns extending far beyond the substitution site. Conflicting red and blue signals across the peptide backbone suggest the model fails to distinguish local chirality effects from global structural noise, leading to incorrect activity prediction. The dispersed attention indicates the model may be confounded by long-range conformational changes or lacks sufficient training on D-Ile substitutions in this sequence context.


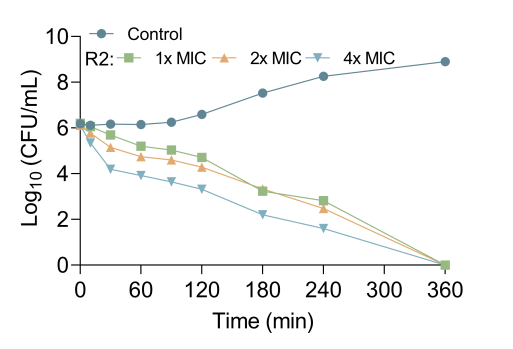


**Figure S6.** Time-killing kinetics curve of *P. aeruginosa* treated with R2.


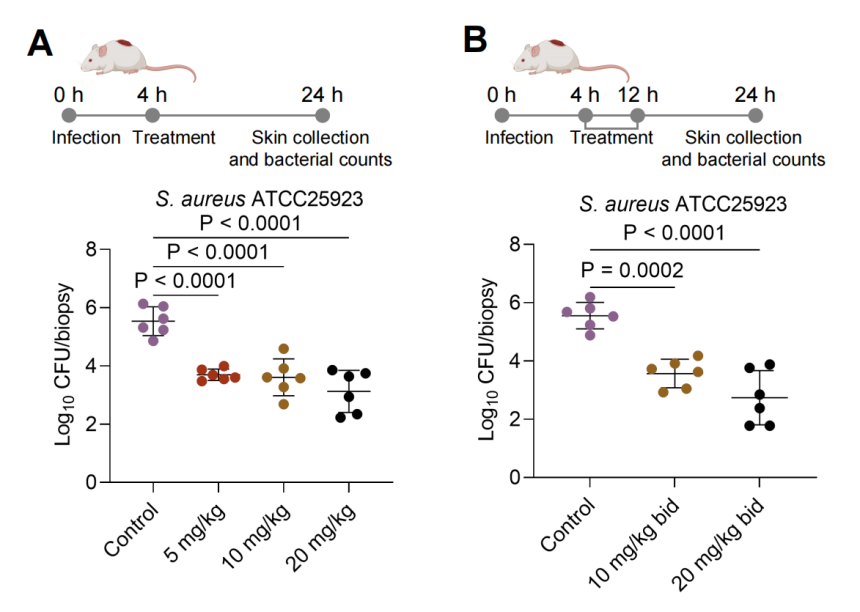


**Figure S7. In vivo antibacterial efficacy of dR2-1.** (**A**) Assessment of bacterial load in skin tissue under a single-dose regimen, n=6. (**B**) Assessment of bacterial load under a multi-dose regimen, n=6. *P* values were determined using one-way ANOVA with Dunnett’s multiple comparison test.


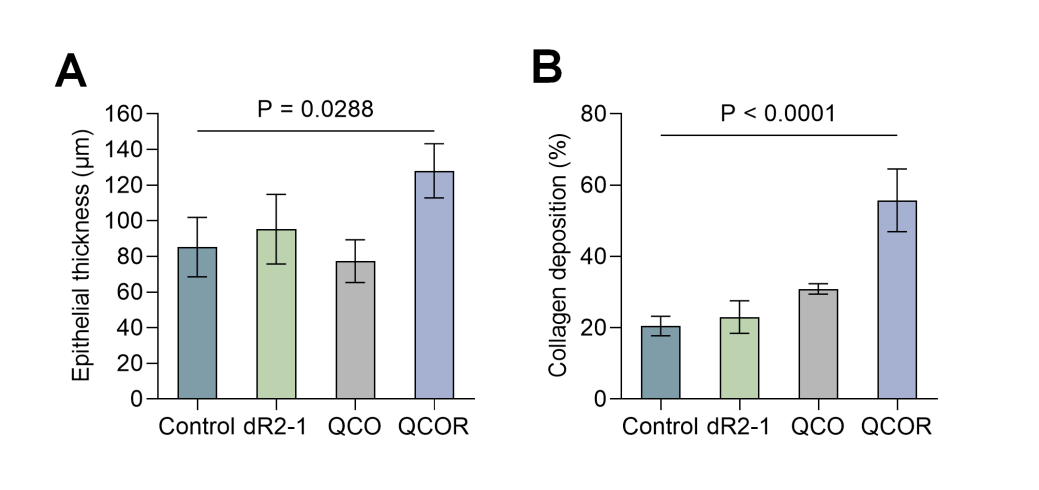


**Figure S8.** Statistical results for epithelial thickness (A) and collagen deposition (B) on day 12 in the in vivo wound healing model.


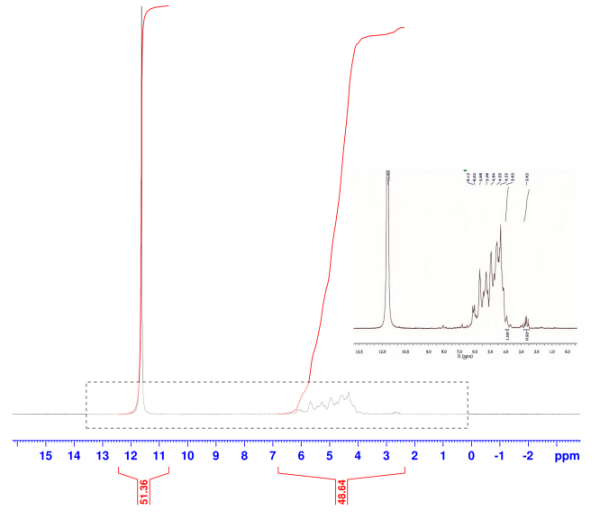


**Figure S9.** ^1^H-nuclear magnetic resonance (NMR) spectrum of quaternized chitosan.


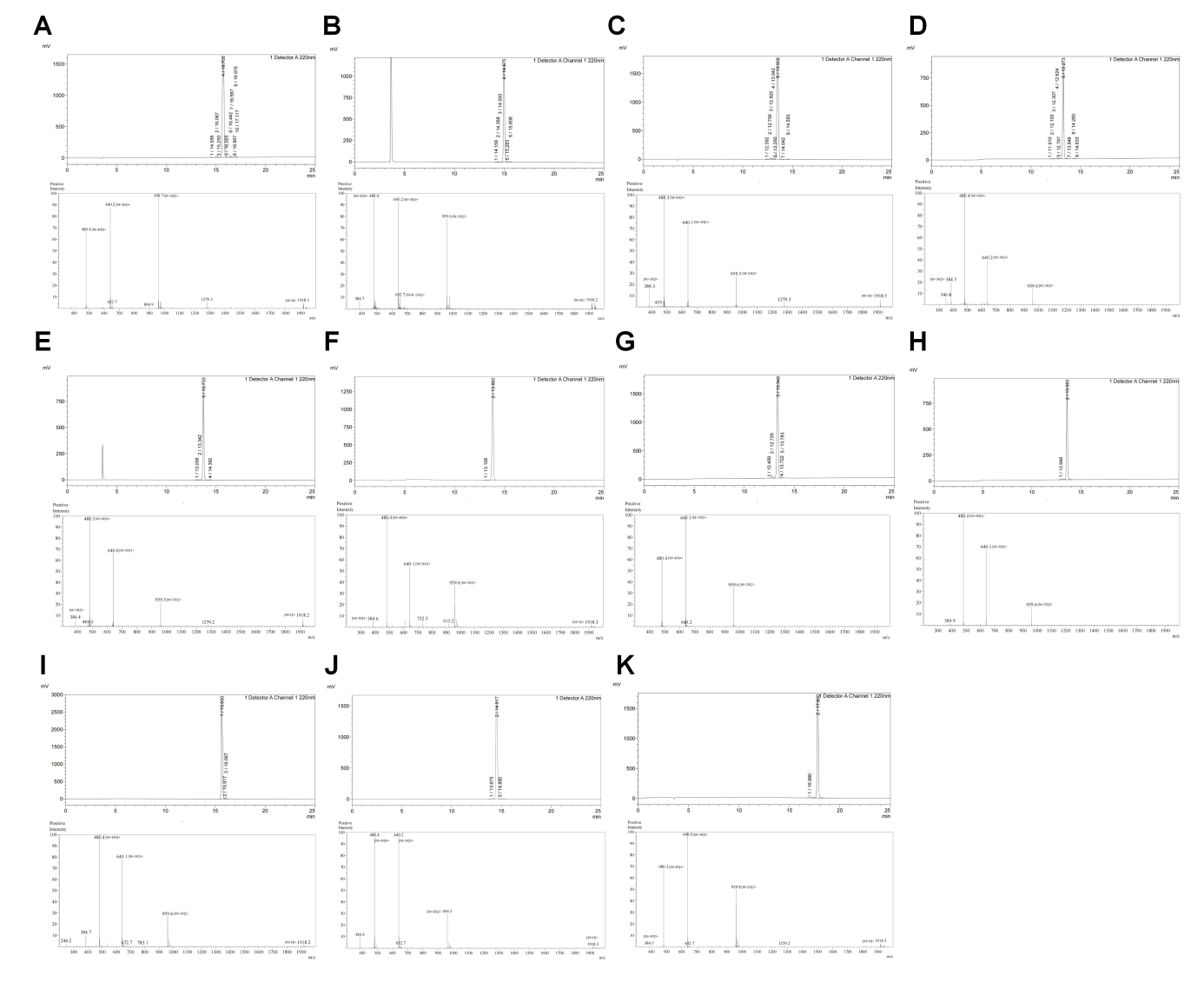


**Figure S10. Molecular characterization of R2 and its derivatives.**

(**A** to **K**) High-performance liquid chromatography (HPLC) (upper) and mass spectrometry (MS) spectra (lower) of R2 (A) and its derivatives dR2-1 to dR2-10 (B to K).


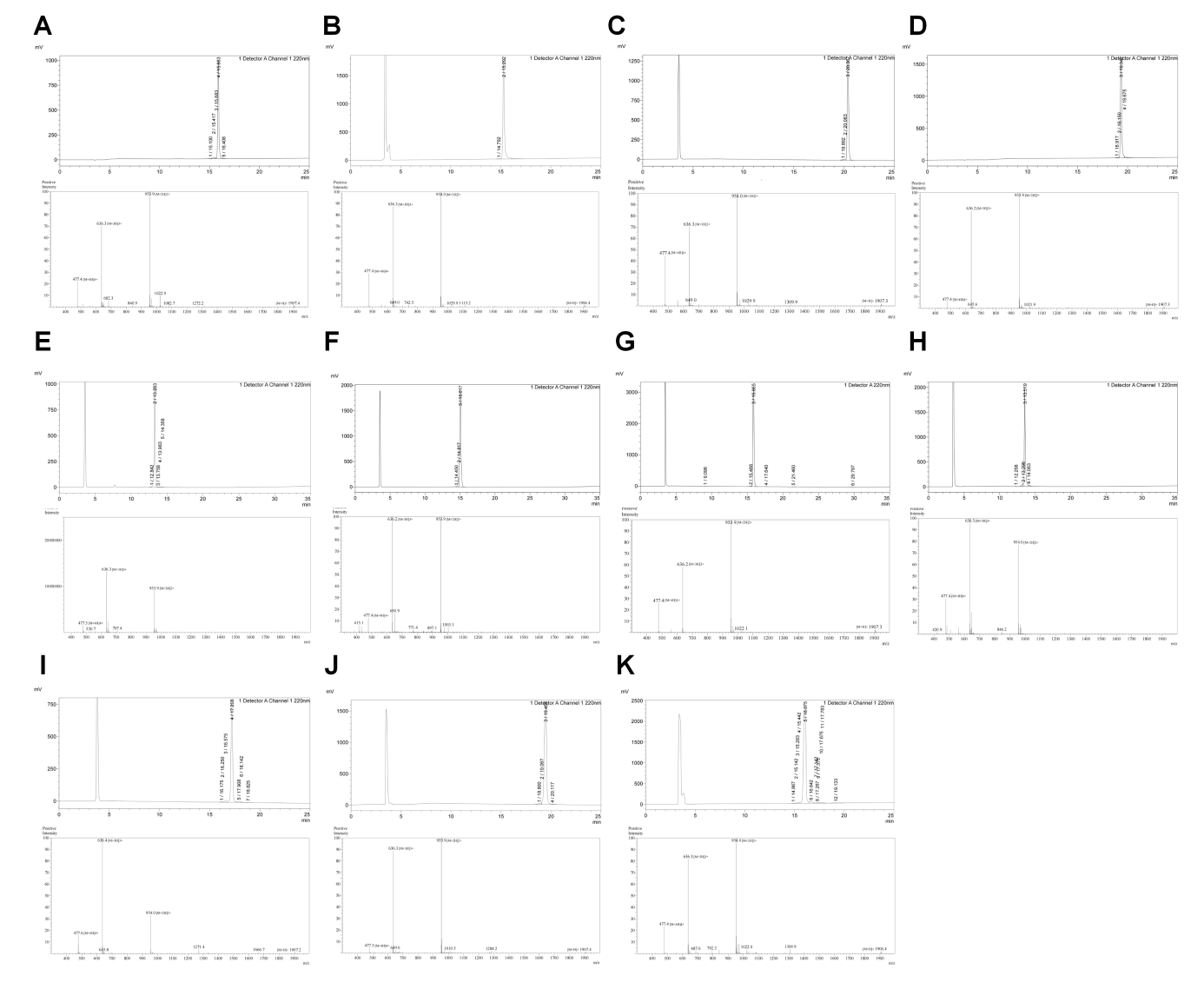


**Figure S11. Molecular characterization of indolicidin and its derivatives.**

(**A** to **K**) High-performance liquid chromatography (HPLC) (upper) and mass spectrometry (MS) spectra (lower) of indolicidin (A) and its derivatives dIndo-1 to dIndo-10 (B to K).

**Table S1.** Protease cleavage rules for coloring SMILES image.

| Protease | Cleavage Specificity |
| --- | --- |
| Trypsin | Cuts immediately after K or R, unless the next residue is P. |
| Chymotrypsin | Cuts after F, Y, W, or L, unless the next residue is P. |
| Elastase | Cuts after A, V, S, G, or T, unless the next residue is P. |
| Enterokinase | Cuts between the K and its N-terminal neighbor in the D-D-D-D-K motif, provided the next residue is not P. |
| Caspase | Cuts between D and the following residue only if the next residue is G, S, or A. |

**Table S2.** Ablation study across three modelling axes:

(1) sequence-only Mamba and other encoders, (2) molecular-image ResNet18 encoders, (3) their multi-modal fusion, and (4) traditional machine learning methods with Morgan fingerprint (MFP) encodings.


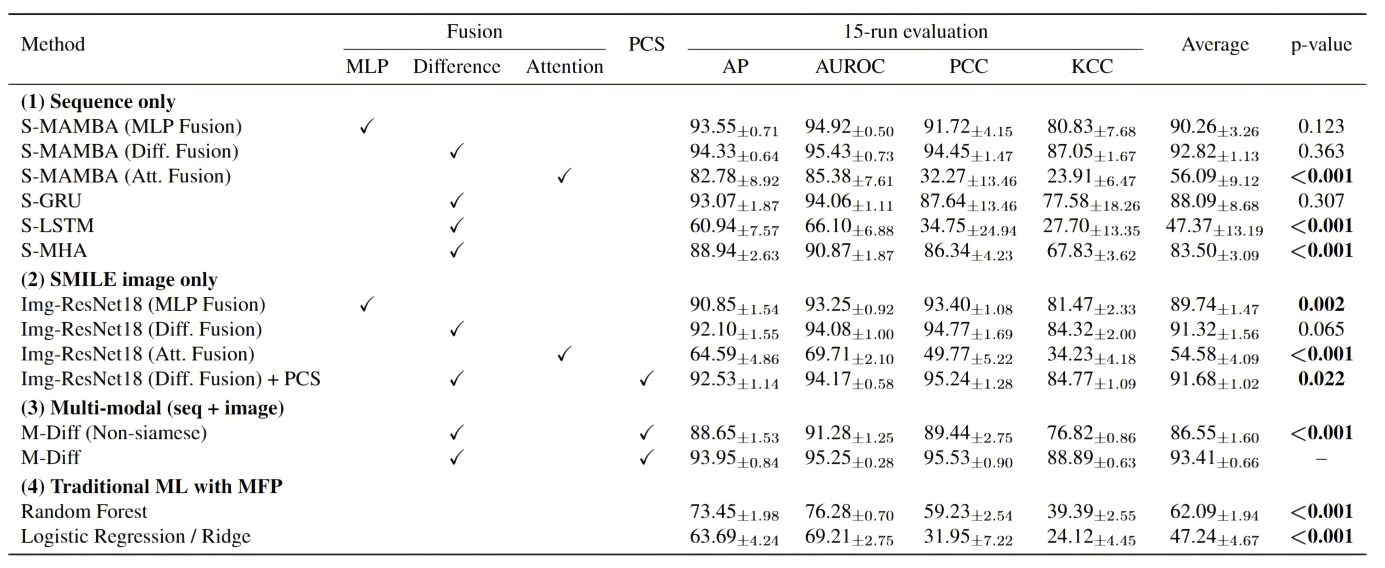


We vary intra-Siamese fusion (MLP, feature *Diff*, or *Attention*), protease cleavage site dyeing (PCS). Average Precision (AP) and Area Under the Receiver Operating Characteristic Curve (AUROC) are classification metrics, while Pearson Correlation Coefficient (PCC) and Kendall's Tau Correlation Coefficient (KCC) are regression metrics. “Average” is the mean of AP, AUROC, PCC, and KCC with their corresponding standard deviations. P-values are computed against the best method (M-Diff) and other methods.

**Table S3.** Comparison of one embedding (ADAPT) versus fused separated embedding approach of PCS dyeing.

| Method | AP | AUROC | PCC | KCC | Average | p-value |
| --- | --- | --- | --- | --- | --- | --- |
| One Embedding (ADAPT) | 93.95±0.84 | 95.25±0.28 | 95.53±0.90 | 88.89±0.63 | 93.41±0.66 | -- |
| Fused Separated Embedding | 93.79±1.40 | 95.07±1.32 | 95.08±1.34 | 87.83±1.49 | 92.94±1.39 | 0.549 |

**Table S4.** Comparison of different initialization strategies for ResNet-18^#^ visual encoder. P-values are computed against the best method (From ImageNet1K).

| Initialization Strategy | AP | AUROC | PCC | KCC | Average | p-value |
| --- | --- | --- | --- | --- | --- | --- |
| No Pretrain | 94.32±1.17 | 95.43±0.93 | 94.45±1.39 | 87.32±1.59 | 92.88±1.27 | 0.510 |
| From ImageMol^*^ | 94.26±0.88 | 95.32±0.72 | 95.68±1.11 | 87.48±2.01 | 93.19±1.18 | 0.765 |
| From ImageNet1K | 93.95±0.84 | 95.25±0.28 | 95.53±0.90 | 88.89±0.63 | 93.41±0.66 | -- |

^#^He, K., Zhang, X., Ren, S. & Sun, J. Deep residual learning for image recognition, 770-778 (IEEE, 2016).

^*^ Zeng, X., Xiang, H., Yu, L. et al. Accurate prediction of molecular properties and drug targets using a self-supervised image representation learning framework. Nat Mach Intell 4, 1004–1016 (2022).

**Table S5.** A comparison of the performance of multiple augmentation methods and UDA on the R2 test set. Demonstrated are the sequence-only model (Mamba), the image-only model (ResNet18), as well as the multi-modal ADAPT. Results are reported as mean±std from 3 different seed runs.


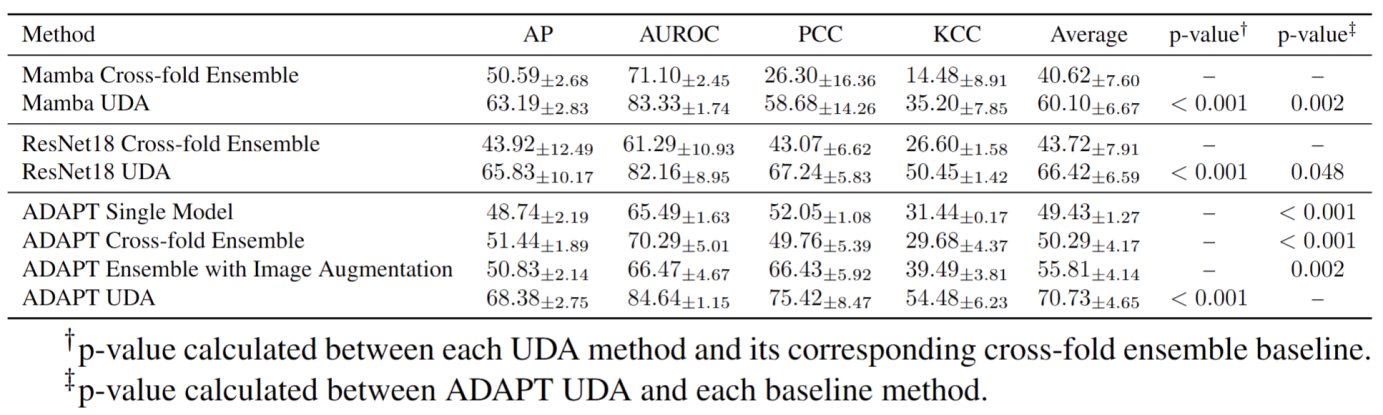


**Table S6.** Sequence and key physicochemical parameters of peptides.

| Peptides | Sequence ^a)^ | Theoretical MW | Measured MW ^b)^ | Purity [%] ^c)^ | T_R_ [min] ^d)^ | Net Charge | Yield [%] | Ranking score ^e)^ |
| --- | --- | --- | --- | --- | --- | --- | --- | --- |
| R2 | KWKIKWPVKWFKML-NH_2_ | 1917.49 | 1917.4 | 96.9 | 15.708 | +6 | 12.48 | N/A ^f)^ |
| dR2-1 | KWKIKWPVKWfKML-NH_2_ | 1917.49 | 1917.6 | 98.1 | 14.975 | +6 | 7.17 | 1.928571 |
| dR2-2 | KwKIKWPVKWFKML-NH_2_ | 1917.49 | 1917.2 | 96.1 | 13.508 | +6 | 26.13 | 1.769059 |
| dR2-3 | KwKIKWPVKWfKML-NH_2_ | 1917.49 | 1917.6 | 96.5 | 13.273 | +6 | 5.27 | 1.768463 |
| dR2-4 | KWKIKWPVKwFKML-NH_2_ | 1917.49 | 1917.2 | 97.8 | 16.192 | +6 | 23.68 | 1.734324 |
| dR2-5 | KWKIKWPVKwfKML-NH_2_ | 1917.49 | 1917.6 | 99.4 | 13.850 | +6 | 2.44 | 1.71816 |
| dR2-6 | kWKIKWPVKWFKML-NH_2_ | 1917.49 | 1917.3 | 96.9 | 13.348 | +6 | 34.39 | 1.712834 |
| dR2-7 | kWKIKWPVKWfKML-NH_2_ | 1917.49 | 1917.6 | 98.1 | 13.585 | +6 | 38.91 | 1.711658 |
| dR2-8 | kwKIKWPVKWfKML-NH_2_ | 1917.49 | 1917.6 | 97.9 | 15.650 | +6 | 39.44 | 1.697938 |
| dR2-9 | KwkIKWPVKWfKML-NH_2_ | 1917.49 | 1917.6 | 99.1 | 14.517 | +6 | 16.48 | 1.665886 |
| dR2-10 | KwkIKWPVKWFKML-NH_2_ | 1917.49 | 1917.0 | 96.5 | 17.800 | +6 | 78.51 | 1.655223 |
| Indolicidin | ILPWKWPWWPWRR-NH_2_ | 1906.32 | 1905.8 | 96.8 | 15.883 | +4 | 22.54 | N/A |
| dIndo-1 | ilpWKWPWWPWRR-NH_2_ | 1906.32 | 1906.0 | 99.4 | 15.292 | +4 | 4.35 | 0.440211 |
| dIndo-2 | ilPWKWPWWPWRR-NH_2_ | 1906.32 | 1906.0 | 98.2 | 20.367 | +4 | 6.12 | 0.428488 |
| dIndo-3 | iLPWKWPWWPWRR-NH_2_ | 1906.32 | 1905.8 | 97.8 | 19.342 | +4 | 8.31 | 0.42652 |
| dIndo-4 | ilpWKWPWWpWRR-NH_2_ | 1906.32 | 1905.9 | 98.3 | 13.283 | +4 | 13.27 | 0.419724 |
| dIndo-5 | iLPWKWPWWpWRR-NH_2_ | 1906.32 | 1905.6 | 95.1 | 15.017 | +4 | 31.2 | 0.417306 |
| dIndo-6 | iLPWKWPWwpWRR-NH_2_ | 1906.32 | 1905.8 | 99.2 | 15.865 | +4 | 34.91 | 0.415132 |
| dIndo-7 | ilPWKWPWWpWRR-NH_2_ | 1906.32 | 1905.9 | 95.2 | 13.519 | +4 | 7.14 | 0.413587 |
| dIndo-8 | iLPWKWPWwpwRR-NH_2_ | 1906.32 | 1906.2 | 95.3 | 17.208 | +4 | 14.54 | 0.410696 |
| dIndo-9 | iLPWKWPWWpwRR-NH_2_ | 1906.32 | 1905.8 | 98.1 | 19.458 | +4 | 18.09 | 0.410446 |
| dIndo-10 | iLPWKWPWwpwrR-NH_2_ | 1906.32 | 1906.0 | 95.1 | 16.075 | +4 | 18.63 | 0.407131 |

^a)^One-letter codes for amino acids. Lowercase letters represent the corresponding D-amino acids.

^b)^Measured molecular weight (MW) was confirmed by MS spectrum.

^c)^Purity of the peptides was measured by HPLC.

^d)^T_R_ (min) represents the retention time measured by HPLC.

^e)^The ranking methodology involves: (1) normalizing classification scores (cls_ft, probability of activity exceeding L-template) and regression scores (reg, predicted MIC) to [0,1]; (2) filtering variants with cls_ft < 0.3; (3) computing synthesis weight as 1 − (D-amino acids / peptide length); and (4) calculating final_score = (1 − reg) × weight to prioritize high activity and synthesis feasibility.

^f)^N/A stands for not applicable.

**Table S7.** Minimal inhibitory concentration (MIC) of dR2-1 and antibiotics.

| Category | Strain | MIC [μg/mL] | | |
| --- | --- | --- | --- | --- |
|  |  | dR2-1 | Polymyxin B | Vancomycin |
| Gram-positive | *E. faecium* 111 | 4 | - | 1 |
|  | *E. faecium* 21 | 8 | - | 1 |
|  | *S. aureus* CMCC26003 | 4 | - | 0.5 |
|  | *S. aureus* 103 | 4 | - | 0.5 |
|  | *S. aureus* ATCC25923 | 8 | - | 0.5 |
|  | *S. aureus* 101 | 8 | - | 1 |
|  | *S. aureus* 129 | 8 | - | 1 |
| Gram-negative | *K. pneumoniae* ATCC BAA-1705 | 4 | 1 | - |
|  | *K. pneumoniae* 209 | 8 | 1 | - |
|  | *A. baumannii* ATCC19606 | 4 | 0.5 | - |
|  | *A. baumannii* 102 | 4 | 0.5 | - |
|  | *A. baumannii* 104 | 4 | 0.5 | - |
|  | *A. baumannii* 106 | 4 | 1 | - |
|  | *A. baumannii* 110 | 4 | 1 | - |
|  | *A. baumannii* 114 | 4 | 0.5 | - |
|  | *P. aeruginosa* PAO1 | 4 | 0.5 | - |
|  | *P. aeruginosa* 116 | 4 | 1 | - |
|  | *E. coli* ATCC25922 | 4 | 0.5 | - |
|  | *E. coli* CMCC44102 | 2 | 1 | - |
|  | *E. coli* CICC21530 | 4 | 1 | - |
|  | *E. coli* 103 | 4 | 0.25 | - |
|  | *E. coli* 166 | 4 | 0.25 | - |
|  | *E. coli* 110 | 8 | 0.5 | - |
|  | *S. enterica* ATCC14028 | 4 | 0.5 | - |
|  | *S. enterica* CMCC50071 | 8 | 1 | - |
| Total ^a)^ |  | 4.7 | - | - |

^a)^Total denotes the geometric mean of MIC values.

**Table S8.** Bacterial strains and resistance information.

| Strain | Description | Source |
| --- | --- | --- |
| *S. aureus* ATCC25923 | Standard strain | Purchased from the China Center of Industrial Culture Collection (CICC) |
| *S. aureus* CMCC26003 | Strain is sensitive to oxacillin | Purchased from the National Center for Medical Culture Collections (CMCC) |
| *S. aureus* 101 | Strain is resistant to oxacillin, erythomycin and clindamycin | Clinical isolate |
| *S. aureus* 103 | Strain is resistant to oxacillin, gentamycin, erythomycin and clindamycin | Clinical isolate |
| *S. aureus* 129 | Strain is resistant to oxacillin, gentamycin, erythomycin and clindamycin | Clinical isolate |
| *S. aureus* 181 | Strain is resistant to erythomycin and clindamycin | Clinical isolate |
| *E. coli* CICC21530 | Strain is sensitive to carbapenem | Purchased from CICC |
| *E. coli* CMCC44102 | Strain is sensitive to carbapenem | Purchased from CMCC |
| *E. coli* ATCC25922 | Standard strain | Purchased from CICC |
| *E. coli* 103 | Strain is sensitive to carbapenem | Clinical isolate |
| *E. coli* 110 | Strain is resistant to ampicillin and cefazolin | Clinical isolate |
| *E. coli* 162 | Strain is resistant to gentamycin and ampicillin | Clinical isolate |
| *E. coli* 166 | Strain is resistant to levofloxacin, gentamycin, polymyxin B and ampicillin | Clinical isolate |
| *E. coli* 208 | Strain is resistant to ceftriaxone, fluoroquinolone, cefuroxime, ampicillin and tetracycline, ESBL (+) | Clinical isolate |
| *A. baumannii* ATCC19606 | Standard strain | Purchased from CICC |
| *A. baumannii* 102 | Strain is resistant to carbapenem, fluoroquinolone, cephalosporin, aminoglycosides and doxycycline | Clinical isolate |
| *A. baumannii* 104 | Strain is resistant to carbapenem, fluoroquinolone, cephalosporin, aminoglycosides and doxycycline | Clinical isolate |
| *A. baumannii* 106 | Strain is resistant to carbapenem, fluoroquinolone, cephalosporin, aminoglycosides and doxycycline | Clinical isolate |
| *A. baumannii* 110 | Strain is resistant to carbapenem, fluoroquinolone, ceftazidime, aminoglycosides and doxycycline | Clinical isolate |
| *A. baumannii* 114 | Strain is resistant to carbapenem, fluoroquinolone, cephalosporin, gentamycin and doxycycline | Clinical isolate |
| *P. aeruginosa* PAO1 | Standard strain | Purchased from CICC |
| *P. aeruginosa* 116 | Strain is resistant to carbapenem | Clinical isolate |
| *K. pneumoniae* ATCC BAA-1705 | Standard strain | Purchased from CICC |
| *K. pneumoniae* 209 | Strain is resistant to carbapenem, fluoroquinolone, aminoglycosides and cephalosporin | Clinical isolate |
| *E. faecium* 111 | Strain is resistant to levofloxacin, gentamycin and ampicillin | Clinical isolate |
| *E. faecium* 21 | Strain is resistant to levofloxacin, gentamycin and ampicillin | Clinical isolate |
| *S. enterica* ATCC14028 | Standard strain | Purchased from CICC |
| *S. enterica* CMCC50071 | Standard strain | Purchased from CMCC |
